# Diagnostic and Prognostic Accuracy of MiRNAs in Pancreatic Cancer: A Systematic Review and Meta‐Analysis

**DOI:** 10.1111/jcmm.70337

**Published:** 2025-01-24

**Authors:** Fatemeh Hasani, Mahdi Masrour, Sina Khamaki, Kimia Jazi, Saba Hosseini, Hadiseh Heidarpour, Mehrad Namazee

**Affiliations:** ^1^ Golestan Research Center of Gastroenterology and Hepatology Golestan University of Medical Sciences Gorgan Iran; ^2^ School of Medicine Tehran University of Medical Sciences Tehran Iran; ^3^ Student Research Committee, Faculty of Medicine Qom University of Medical Sciences Qom Iran; ^4^ School of Medicine Shiraz University of Medical Sciences Shiraz Iran

**Keywords:** biomarker, diagnosis, microRNAs, pancreatic cancer, prognosis

## Abstract

Pancreatic cancer (PC) remains a significant contributor to global cancer mortality, with limited effective diagnostic and prognostic tools. MicroRNAs (miRNAs) have emerged as promising biomarkers for PC diagnosis and prognosis. A comprehensive literature search was conducted in PubMed, Web of Science, and Scopus. Studies reporting sensitivity, specificity or area under the curve (AUC) for miRNAs in PC diagnosis, as well as hazard ratios (HRs) for survival evaluations, were included. Data extraction and quality assessment followed PRISMA guidelines. Meta‐analyses were conducted using appropriate statistical methods. The protocol is registered in PROSPERO. Diagnostic analysis included 290 evaluations, revealing an overall AUC of 0.8226 for PC diagnosis. Subgroup analyses showed varying accuracies, with blood and tissue specimens yielding higher AUC values. Promising miRNAs with AUC values above 0.8 included miR‐320, miR‐1290, miR‐93, miR‐25, miR‐451, miR‐20, miR‐21, miR‐223 and miR‐122. Prognostic analysis encompassed 46 studies, indicating significant associations between miRNA expression and overall survival (OS) and progression‐free survival (PFS). The combined HR for studies reporting OS HRs higher than one was 1.7613 (95% CI: 1.5394–2.0152, *p* < 0.0001; *I* ^2^ = 81.7%). Notable miRNAs with prognostic significance included miR‐10, miR‐21 and miR‐221. Studies reporting OS HRs less than one had a pooled HR of 0.6805 (95% CI: 0.5862–0.7901, *p* < 0.0001; *I* ^2^ = 65.4%). MiRNAs hold promise as diagnostic and prognostic biomarkers for PC. Blood and tissue specimens offer superior diagnostic accuracy, and several miRNAs show potential for predicting patient outcomes.

## 1 Introduction

Pancreatic cancer (PC) stands as a leading cause of cancer mortality worldwide. Regions with the greatest prevalence of PC include North America, Europe and Australia [1]. PC currently accounts for the third major cause of cancer‐associated mortality in the United States. It is estimated that 62,200 new cases are diagnosed annually in the United States resulting in 48,800 deaths [2]. Despite significant advancements in medical and surgical interventions for PC over the past decades, the 5‐year survival rate for PC remains discouraging. The majority of PC patients are diagnosed at an advanced clinical stage, as they typically have no symptoms until the cancer has already metastasized to distant organs [3, 4, 5, 6].

So far, the serum carbohydrate antigen 19–9 (CA 19–9) has been used as a tumour marker for evaluating clinical treatment effectiveness in pancreatic cancer [7, 8]. Despite CA 19–9 being the only FDA‐approved marker in PC, there are some limitations associated with it, including ineffectiveness, low sensitivity, and low specificity. Other tumour markers used in PC diagnosis, including Carcinoembryonic antigen (CEA) and Cancer antigen 125 (CA125), demonstrated less effectiveness compared to CA 19–9. However, they are still routinely utilised by some oncologists to monitor the extent of disease and response to therapy, when there is an elevation in their levels from the initial baseline [7, 9]. According to GLOBOCAN, there were 511,000 new cases of pancreatic cancer and 467,000 deaths in 2022. With one of the poorest prognoses, pancreatic cancer is the sixth leading cause of cancer mortality among both sexes combined [10]. Due to the challenges associated with early detection and the absence of effective treatment options, the 5‐year survival rate for pancreatic cancer has remained below 10% over the past two decades [11, 12, 13]. Only 15%–20% of pancreatic cancer cases are currently diagnosed at a stage that allows for the possibility of curative surgery. Early detection of pancreatic cancer poses significant challenges, as PanIN lesions are microscopic and usually identified in resected specimens obtained for other clinical reasons; they typically remain undetectable through preoperative imaging [14, 15, 16].

Hence, a key area of focus in PC research involves the identification of novel biomarkers detectable in easily accessible samples like blood and other bodily fluids having potential for early‐stage diagnosis, prognosis and even surveillance of Pancreatic cancer [17].

MicroRNAs (miRNAs) are a group of endogenous, small, noncoding RNA molecules ranging from 20 to 24 nucleotides. They serve as posttranscriptional regulators, suppressing the expression of one or more target genes by binding to the 3′‐untranslated regions (UTRs) of mRNAs in most cases [18, 19, 20]. Nevertheless, there have been reports of miRNA interaction with other binding regions such as the 5′‐UTR, coding sequence, and gene promoters as well [21, 22, 23].

MiRNAs serve as critical biomarkers across various cancers due to their unique regulatory roles in gene expression and tumour biology. downregulation of miR‐371b‐5p leads to increased expression of target genes like Smad2 and LEF1, which are involved in pathways promoting cancer progression and chemoresistance in T‐LBL (T‐lymphoblastic lymphoma) [24]. The miR‐17‐92 cluster, which includes miRs‐17, ‐18a, ‐19a, ‐20a, ‐19b and ‐92a, has been shown to promote tumour progression and is commonly overexpressed in several cancers. Specifically, elevated levels of this cluster have been observed in cancers such as small‐cell lung cancer, colorectal adenoma organoids, hepatocellular carcinoma, thyroid cancer, colon cancer and renal cell carcinoma [25, 26, 27, 28, 29, 30]. Moreover, plasma samples from breast cancer patients show significantly elevated levels of miR‐146a [31]. Another study showed that miR‐21 ‐141, and ‐221 levels in blood plasma are elevated in prostate cancer patients compared to healthy individuals [32]. miR‐21 promotes tumour growth and drug resistance in pancreatic ductal adenocarcinoma [33].

Increasing studies indicated that tumour‐specific circulating miRNAs exhibit a stable presence in numerous human body fluids, such as serum, urine, gastric juice, synovial fluid and amniotic fluid. These miRNAs function as innovative, noninvasive and optimised biomarkers for diagnosing various malignancies, including PDAC [19, 34]. For instance, Cote et al. found increased expression of miRNA‐10b, ‐155 and 106b in plasma as a highly accurate diagnostic tool for PDAC [35]. In another investigation conducted by Liu et al., the expression levels of 7 miRNAs (miR‐20a, miR‐21, miR‐24, miR‐25, miR‐99a, miR‐185 and miR‐191) showed significant differences between pancreatic cancer patients and control subjects. Implementation of this panel of 7 miRNA‐based biomarkers for pancreatic cancer diagnosis, resulted in an accuracy of 83.6%, which was higher than CA19‐9, that was 56.4% [36, 37]. In another study by Cote et al. The differential expression of 9/10 miRNAs in plasma, namely miR‐10b, ‐30c, ‐106b, ‐132, ‐155, ‐181a, ‐181b, ‐196a and ‐212, and 7/20 in bile (excluding miR‐21, ‐132, and ‐181b), was observed. Among these, five miRNAs (miR‐10b, ‐155, ‐106b, ‐30c, and ‐212) had remarkable accuracy in distinguishing PDAC [35].

The first indications of miRNA's potential prognostic significance came from Takamizawa et al., who discovered a consistent decrease in let‐7 expression levels among patients with lung cancer, correlating significantly with worse survival outcomes following tumour resection [17, 38]. Moreover, several miRNAs have also been identified as prognostic indicators for clinical outcomes in pancreatic adenocarcinoma [39, 40]. As an example, Elevated levels of exosomal miR‐483‐3p were associated with poorer survival outcomes in patients with PDAC. Furthermore, serum exosomal miR‐483‐3p was demonstrated to act as a prognostic factor for PDAC [41]. In a study conducted by Dillhoff et al., miRNA‐21 was significantly overexpressed in PC as detected by in situ hybridization. Its robust expression was predictive of limited survival in patients with node‐negative disease [22]. Also, a large clinical investigation, including 686 patients, revealed a significant correlation between elevated miR‐21 levels and shorter overall survival in PDAC tumours. Additionally, it demonstrated a significant association with tumour size and lymph node metastasis [42].

Hence the present Systematic review and meta‐analysis was conducted to assess the diagnostic and prognostic accuracy of miRNAs in pancreatic cancer based on the published literatures.

## 2 Methods

We performed a systematic review and meta‐analysis in accordance with the PRISMA standards [43]. Our systematic review and meta‐analysis protocol has been registered at PROSPERO with the registration number CRD42023481890.

### 2.1 Literature Search

An extensive search in PubMed, Web of Science (ISI), and Scopus was conducted on December 2nd, 2023, to find English articles without any limitation on publication year. Databases were searched using medical subject headings (MeSH) terms and free keywords, including “microRNA” and “pancreatic cancer” and their expansions. The Table S1 provide the search query.

### 2.2 Selection Criteria

The current study included peer‐reviewed original research that provided the sensitivity, specificity, or area under the curve (AUC) values of microRNAs in the diagnosis of pancreatic cancer, as well as their correlation with prognosis as measured by overall survival (OS), progression‐free survival (PFS), disease‐free survival (DFS), recurrence‐free survival (RFS) and event‐free survival. We regarded case–control and cohort human studies as eligible for inclusion in our review. The included research investigations were conducted either prospectively or retrospectively, and they employed samples from both pancreatic cancer patients and controls. In diagnostic accuracy investigations, microRNAs should have been compared to an appropriate reference control to determine sensitivity, specificity and AUC. There were no eligibility restrictions based on the healthcare settings in which the study was conducted or on the total number of individuals included in the study. Non‐English research, studies on animal models, letters, comments, reviews, editorials, conference abstracts, case reports and case series were deemed unsuitable and hence eliminated from the study.

After removing any duplicates, FH and SK reviewed the remaining identified papers and determined their eligibility using the previously defined inclusion and exclusion criteria. After establishing a list of articles that met the qualifying criteria, both authors separately conducted a thorough assessment of the complete texts of the studies. During the review process, any issues that developed were efficiently handled by reaching a consensus.

### 2.3 Data Extraction

Two reviewers (FH and SK) separately extracted data from each of the included studies into a dedicated computer spreadsheet. When available, the following information was extracted from each study: authors, publication year, specimen type, sample size, type of pancreatic cancer, control population, microRNA name, change in microRNA levels in patients compared to the control group, diagnostic or prognostic performance measures, such as sensitivity, specificity, AUC with corresponding 95% confidence interval (CI) and *p*‐value, and mean, median, and hazard ratio (HR) for survival outcomes with corresponding 95% CI and *p*‐value. Disagreements were addressed by a third reviewer (HH).

### 2.4 Quality Assessment

The Newcastle‐Ottawa Scale (NOS) was used to assess the quality of included studies in cohort and case–control studies [44]. Two reviewers (FZ and KJ) independently evaluated the quality of each study using predetermined criteria. Any differences in the quality rating were addressed through discussion or contact with a third reviewer. NOS has three basic areas of bias: selection, comparability and outcome. Scores of 7 and above, 2–6, and 1 and below were classified as “good,” “fair,” and “poor,” respectively.

### 2.5 Statistical Analysis

The inverse variance approach was used to meta‐analyse the AUC values given in the studies. We also utilised the bivariate random effect model established by Reitsma et al. to integrate the studies that provided diagnostic specificity and sensitivity. When calculating the outcomes, the model considers the two variables' interdependence [45]. This model also computes the summary receiver operating characteristic (sROC) curve and the AUC, which measure the diagnostic accuracy. Since there was expected to be variability and heterogeneity among the research examined, the random effects model was adopted. We used the inverse variance approach on HR values to do a meta‐analysis of prognostic values, which were presented as HRs. The random effects model was utilised to account for the observed heterogeneity in the reported values. HR less than one and HR more than one were separated into two separate groups.

The standard error of the AUCs and HRs for meta‐analysis was determined using the 95% CI. If the AUC's CI was not available, the AUC value and sample size (the Hanley and McNeil method) were used to estimate the standard error [46, 47]. The study used *I* ^2^ and T_2_ estimator statistics to estimate research heterogeneity. To investigate the heterogeneity further, a subgroup analysis was performed using the sample types. The statistical analyses and visualisations were performed in R version 4.2.2 (R Core Team [2021], Vienna, Austria) using the “meta” and “mada” packages [48, 49]. Statistical significance was determined by an *I* ^2^ value exceeding 50% and a *p*‐value below 0.05.

## 3 Results

**FIGURE 1:**
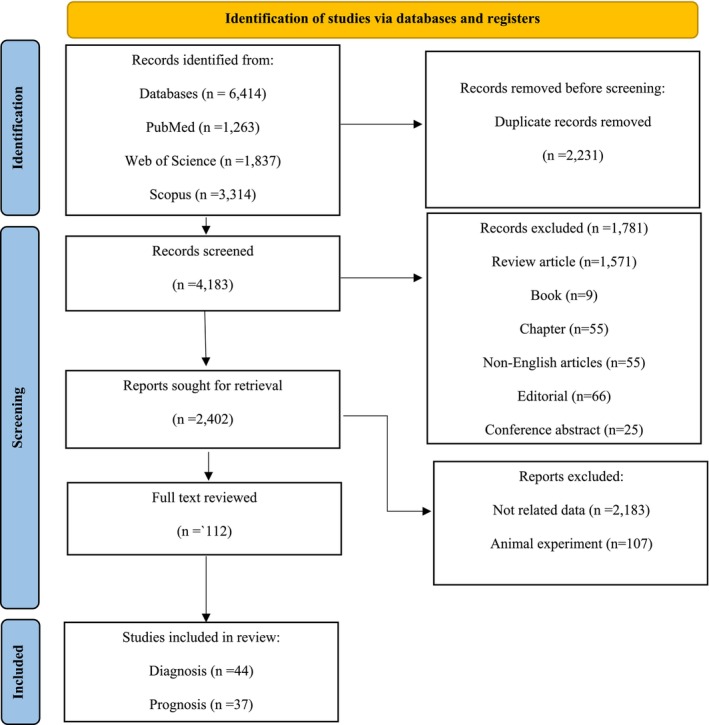
PRISMA flow diagram.

Our search retrieved a total of 6414 records from PubMed (*n* = 1263), Web of Science (*n* = 1837), and SCOPUS (*n* = 3314). After removing duplicates and excluding studies that did not meet our criteria, we included 81 studies in our analysis. These studies involved 5630 pancreatic cancer patients and 4380 controls in diagnostic assessments, and 3349 pancreatic cancer patients and 927 controls in prognostic assessments. A PRISMA flow chart illustrating the study selection process is shown in Figure 1. Detailed information about the included studies can be found in Tables S2 and S3. All included studies were rated as “good” or “very good” based on their NOS quality assessment scores, as detailed in Tables S4 and S5.

### 3.1 Diagnostic Accuracy of MicroRNAs

#### 3.1.1 Overall Findings

MicroRNA AUC values for PC diagnosis were reported in 290 evaluations. Using the random effect model, the combined AUC value was 0.8226 (95% CI: 0.8182–0.8270, *p* < 0.0001, *I* ^2^ = 99.0%). The evaluations were divided into five subgroups based on the type of specimen used to assess microRNA expression: Blood, Saliva, Tissue, Pancreatic juice and Urine (Figure S1). With 232 evaluations, the AUC for the blood specimen subgroup was 0.7926 (95% CI: 0.7873–0.7980; *I* ^2^ = 99.2%). The tissue specimen subgroup, comprising 46 evaluations, had a pooled AUC of 0.8433 (95% CI: 0.8209–0.8657; *I* ^2^ = 96.7%). For the subgroup of saliva specimens with five examinations, the pooled AUC was 0.7202 (95% CI: 0.5233–0.9171; *I* ^2^ = 95.0%). The pancreatic juice subgroup, comprising 6 evaluations, had a pooled AUC of 0.7235 (95% CI: 0.6373–0.8096; *I* ^2^ = 54.1%). Urine samples were only used in one trial, which had an AUC of 0.9000 for PC diagnosis. A statistically significant difference was seen when comparing the subgroups (*p* < 0.0001), demonstrating that the PC's diagnostic power varied according to the type of specimen. The characteristics of included studies in case of diagnosis is provided in Table S2.

The Reitsma bivariate model acquired a cumulative sensitivity of 0.732 (95% CI: 0.713–0.750, *p* < 0.001) and a pooled specificity of 0.748 (95% CI: 0.729–0.766, *p* < 0.001) for diagnostic evaluations reporting sensitivity and specificity. The estimated *I* ^2^ value using the Holling sample size unadjusted approaches was 60%–74.6%. This analysis included 165 diagnostic evaluations using blood specimens, 46 diagnostic evaluations using tissue specimens, 5 evaluations using saliva specimens, and 1 evaluation using urine specimens. The computed AUC for all evaluations combined was found to be 0.803 after the summary ROC curve was generated. With 165 evaluations, microRNAs in blood specimens showed a sensitivity of 0.700 (95% CI: 0.679–0.720, *p* < 0.001) and a cumulative specificity of 0.745 (95% CI: 0.724–0.765, *p* < 0.0001). Using the Holling sample size unadjusted techniques, the estimated *I* ^2^ value was between 55.7% and 72.1%. For the blood specimen investigations, the sROC‐generated AUC was 0.782. The 42 evaluations on the diagnostic accuracy of microRNAs in tissue samples exhibited a cumulative sensitivity of 0.827 (95% CI: 0.792–0.858, *p* < 0.001) and a pooled specificity of 0.786 (95% CI: 0.745–0.821, *p* < 0.001). The estimated *I* ^2^ value using the Holling unadjusted approaches was 67.5%–75.8%. The sROC‐generated AUC for tissue specimen studies was 0.872. The five evaluations on the diagnostic accuracy of microRNAs in saliva samples exhibited a cumulative sensitivity of 0.844 (95% CI: 0.723–0.918, *p* < 0.001) and a pooled specificity of 0.457 (95% CI 0.306–0.617, *p* = 0.603). The estimated *I* ^2^ value using the Holling unadjusted approaches was 0%–0%. The sROC‐generated AUC for saliva specimen studies was 0.721. Four evaluations on the diagnostic accuracy of microRNAs in Pancreatic juice samples exhibited a cumulative sensitivity of 0.671 (95% CI: 0.564–0.763, *p* = 0.002) and a pooled specificity of 0.839 (95% CI 0.725–0.911, *p* < 0.001). The estimated *I* ^2^ value using the Holling unadjusted approaches was 48.7%–49.2%. The sROC‐generated AUC for saliva specimen studies was 0.850. The only study on the diagnostic accuracy of microRNAs in urine samples found a sensitivity of 0.902 and specificity of 0.833, with a reported AUC of 0.9.

#### 3.1.2 Promising MicroRNAs

**FIGURE 2:**
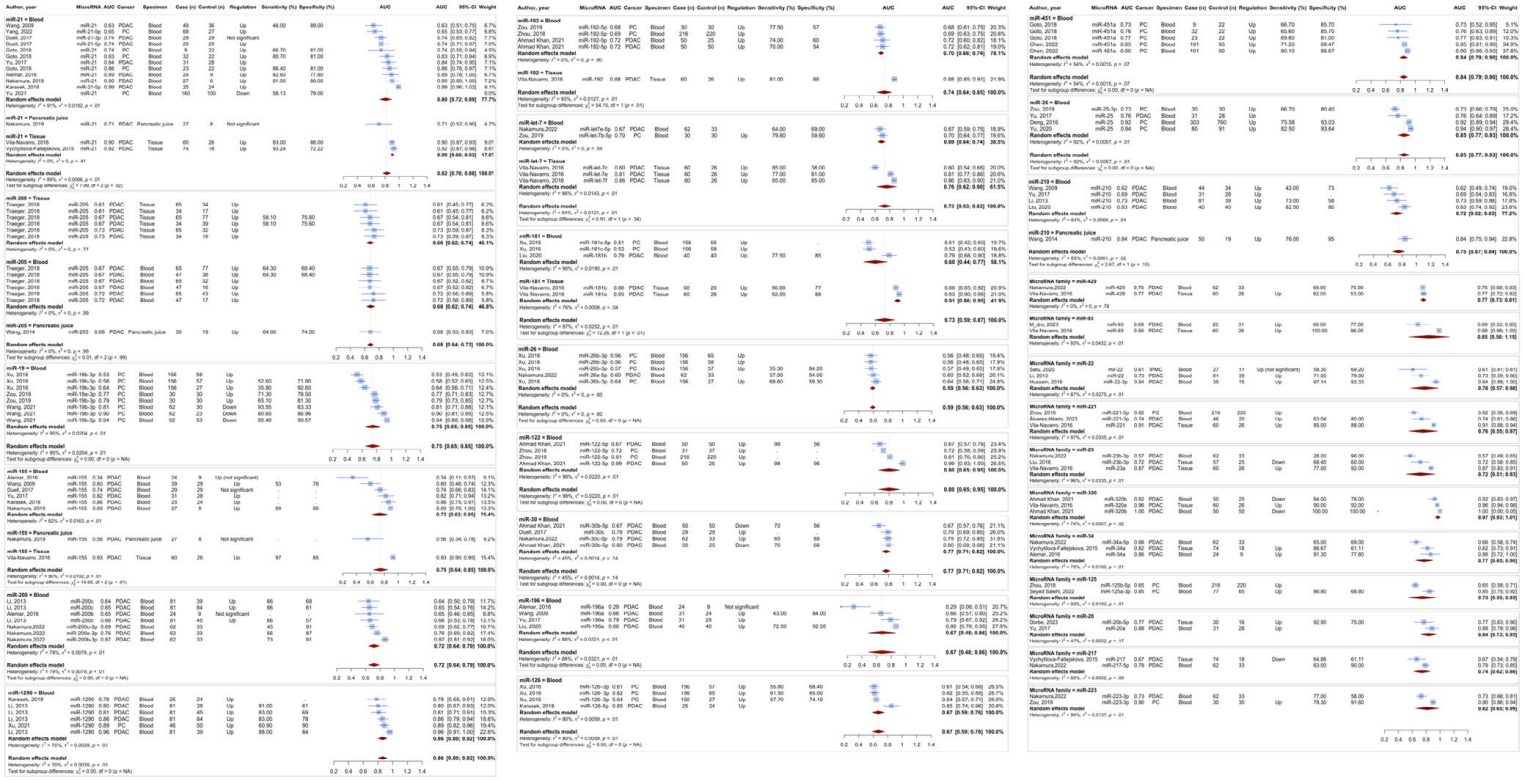
Diagnostic accuracy of microRNAs for pancreatic cancer. Combined area under the curve (AUC) value; across 290 evaluations, the overall combined AUC for PC diagnosis using microRNAs was 0.8226 (95% CI: 0.8182–0.8270). Subgroup analysis by specimen type; blood: 0.7926 (95% CI: 0.7873–0.7980), tissue: 0.8433 (95% CI: 0.8209–0.8657), saliva: 0.7202 (95% CI: 0.5233–0.9171), pancreatic juice: 0.7235 (95% CI: 0.6373–0.8096), urine: 0.9000 (one evaluation). Sensitivity and specificity; overall sensitivity: 0.732 (95% CI: 0.713–0.750), overall specificity: 0.748 (95% CI: 0.729–0.766), blood specimen: sensitivity: 0.700 (95% CI: 0.679–0.720), specificity: 0.745 (95% CI: 0.724–0.765), tissue specimen: sensitivity: 0.827 (95% CI: 0.792–0.858), specificity: 0.786 (95% CI: 0.745–0.821). MicroRNAs with high diagnostic accuracy; miR‐320: AUC > 0.9 (pooled AUC: 0.9694), miR‐1290: AUC 0.8632, miR‐93: AUC 0.8534, miR‐25: AUC 0.8485, miR‐451: AUC 0.8439, miR‐20: AUC 0.8368, miR‐21: AUC 0.8212, miR‐223: AUC 0.8186, miR‐122: AUC 0.8009. Extensively studied microRNAs; miR‐21: combined AUC: 0.8212 (14 evaluations), blood samples: AUC 0.8049 (11 evaluations), tissue samples: AUC 0.9031 (2 evaluations), pancreatic juice: AUC 0.7100 (1 evaluation).

We conducted a meta‐analysis on microRNAs that were the subject of two or more different studies reporting on their diagnostic accuracy. The results are succinctly presented in Table 1 and Figure 2. After sorting the findings of the meta‐analyses by the pooled AUC, it was found that one specific microRNA, miR‐320, had an AUC value more than 0.9 (pooled AUC [95% CI] 0.9694 [0.9335–1.0054]). Out of the microRNAs meta‐analysed, eight had an AUC greater than 0.8. These microRNAs were miR‐1290 (pooled AUC [95% CI] 0.8632 [0.8015–0.9249]), miR‐93 (pooled AUC [95% CI] 0.8534 [0.5553–1.1515]), miR‐25 (pooled AUC [95% CI] 0.8485 [0.7675–0.9294]), miR‐451 (pooled AUC [95% CI] 0.8439 [0.7920–0.8958]), miR‐20 (pooled AUC [95% CI] 0.8368 [0.7252–0.9485]), miR‐21 (pooled AUC [95% CI] 0.8212 [0.7628–0.8796]), miR‐223 (pooled AUC [95% CI] 0.8186 [0.6511–0.9860]), miR‐122 (pooled AUC [95% CI] 0.8009 [0.6530–0.9487]). When the meta‐analysis findings were sorted by the sROC‐generated AUC, it was found that one microRNA, miR‐320, had an AUC greater than 0.9 (AUC 0.965). Nine microRNAs had an sROC‐generated AUC greater than 0.8. These microRNAs were miR‐451 (AUC 0.885), miR‐22 (AUC 0.885), miR‐25 (AUC 0.871), miR‐181 (AUC 0.870), miR‐1290 (AUC 0.856), miR‐155 (AUC 0.855), miR‐19 (AUC 0.839), miR‐21 (AUC 0.822) and miR‐let‐7 (AUC 0.803).

**TABLE 1:** Results of the meta‐analysis summarised for microRNAs with multiple evaluations.

MicroRNA	AUC evaluations	Pooled AUC [95% CI]	AUC *p*	*I* ^2^	Sensitivity/specificity evaluations	Pooled sensitivity [95% CI]	Sensitivity *p*	Pooled specificity [95% CI]	Specificity *p*	sROC calculated AUC	Holling unadjusted *I* ^2^
miR‐320	3	0.9694 [0.9335; 1.0054]	< 0.0001	73.90%	3	0.921 [0.739; 0.980]	0.001	0.928 [0.657; 0.989]	0.009	0.965	55.8%–78.9%
miR‐1290	6	0.8632 [0.8015; 0.9249]	< 0.0001	70.10%	5	0.800 [0.712; 0.867]	< 0.001	0.778 [0.662; 0.863]	< 0.001	0.856	43.2%–44%
miR‐93	2	0.8534 [0.5553; 1.1515]	< 0.0001	92.80%							
miR‐25	4	0.8485 [0.7675; 0.9294]	< 0.0001	91.80%	3	0.744 [0.653; 0.818]	< 0.001	0.911 [0.822; 0.958]	< 0.001	0.871	64.9%–81.1%
miR‐451	5	0.8439 [0.7920; 0.8958]	< 0.0001	54.30%	5	0.733 [0.664; 0.793]	< 0.001	0.872 [0.824; 0.908]	< 0.001	0.885	0%–0%
miR‐20	2	0.8368 [0.7252; 0.9485]	< 0.0001	47.10%							
miR‐21	14	0.8212 [0.7628; 0.8796]	< 0.0001	88.70%	9	0.774 [0.653; 0.862]	< 0.001	0.808 [0.753; 0.853]	< 0.001	0.822	59.9%–75.3%
miR‐223	2	0.8186 [0.6511; 0.9860]	< 0.0001	93.60%							
miR‐122	4	0.8009 [0.6530; 0.9487]	< 0.0001	98.60%							
miR‐22	3	0.7763 [0.5725; 0.9801]	< 0.0001	87.30%	3	0.804 [0.434; 0.956]	0.099	0.835 [0.626; 0.938]	0.004	0.885	67.6%–85.1%
miR‐34	3	0.7741 [0.6458; 0.9023]	< 0.0001	79.30%	3	0.815 [0.609; 0.926]	0.005	0.675 [0.544; 0.784]	0.01	0.7	57.6%–71.3%
miR‐429	2	0.7690 [0.7274; 0.8106]	< 0.0001	0.00%							
miR‐30	4	0.7656 [0.7098; 0.8213]	< 0.0001	44.80%	3	0.700 [0.612; 0.774]	< 0.001	0.714 [0.478; 0.872]	0.074	0.726	31.1%–31.9%
miR‐221	3	0.7595 [0.5475; 0.9715]	< 0.0001	97.10%							
miR‐210	5	0.7511 [0.6651; 0.8371]	< 0.0001	64.50%	4	0.693 [0.501; 0.836]	0.049	0.751 [0.594; 0.862]	0.003	0.786	83.4%–85.2%
miR‐19	8	0.7471 [0.6452; 0.8491]	< 0.0001	94.80%	7	0.719 [0.534; 0.851]	0.022	0.809 [0.714; 0.877]	< 0.001	0.839	80.1%–85%
miR‐155	8	0.7456 [0.6386; 0.8527]	< 0.0001	89.70%	3	0.856 [0.481; 0.974]	0.06	0.829 [0.708; 0.906]	< 0.001	0.855	87%–91.9%
miR‐125	2	0.7455 [0.5456; 0.9453]	< 0.0001	92.90%							
miR‐192	5	0.7423 [0.6382; 0.8464]	< 0.0001	92.70%	4	0.755 [0.673; 0.821]	< 0.001	0.647 [0.471; 0.791]	0.1	0.77	70.1%–71.2%
miR‐217	2	0.7412 [0.6217; 0.8607]	< 0.0001	66.10%							
miR‐let‐7	5	0.7326 [0.6327; 0.8325]	< 0.0001	94.30%	5	0.784 [0.696; 0.852]	< 0.001	0.673 [0.498; 0.811]	0.053	0.803	55.6%–58.2%
miR‐181	5	0.7285 [0.5855; 0.8714]	< 0.0001	97.50%	3	0.871 [0.765; 0.933]	< 0.001	0.831 [0.736; 0.896]	< 0.001	0.87	44%–45.3%
miR‐23	3	0.7210 [0.5071; 0.9349]	< 0.0001	95.60%	3	0.581 [0.275; 0.835]	0.623	0.880 [0.521; 0.980]	0.041	0.796	77.2%–77.5%
miR‐200	7	0.7158 [0.6380; 0.7936]	< 0.0001	79.20%	6	0.617 [0.544; 0.685]	0.002	0.773 [0.619; 0.877]	0.001	0.691	56.3%–57.3%
miR‐205	13	0.6802 [0.6404; 0.7200]	< 0.0001	0.00%	5	0.620 [0.560; 0.677]	< 0.001	0.719 [0.660; 0.771]	< 0.001	0.646	0%–0%
miR‐196	4	0.6741 [0.4833; 0.8648]	< 0.0001	87.50%							
miR‐126	4	0.6707 [0.5857; 0.7557]	< 0.0001	80.00%	3	0.583 [0.535; 0.630]	0.001	0.66 [0.568; 0.742]	0.001	0.626	0%–0%
miR‐26	5	0.5910 [0.5552; 0.6269]	< 0.0001	0.00%	3	0.467 [0.260; 0.686]	0.776	0.801 [0.568; 0.925]	0.015	0.682	0%–0%

The microRNA that has been extensively studied for diagnosing PC cases from controls was miR‐21. There have been 14 evaluations reporting on its AUC and 9 evaluations reporting on its sensitivity and specificity. The meta‐analysis performed on AUC reporting studies yielded a combined AUC of 0.8212 (95% CI: 0.7628–0.8796; *I* ^2^ = 88.7%), indicating statistical significance (*p* < 0.0001). Out of the 14 assessments conducted, 11 were performed on blood samples and had a combined AUC value of 0.8049 (95% CI: 0.7165–0.8932; *I* ^2^ = 91.0%). Two evaluations were conducted on tissue samples and had a combined AUC value of 0.9031 (95% CI: 0.8768–0.9293; *I* ^2^ = 0.0%). The other evaluation was conducted on pancreatic juice samples and had an AUC value of 0.7100. The meta‐analysis performed on 9 evaluations of miR‐21 sensitivity and specificity yielded a combined sensitivity of 0.774 (95% CI: 0.653–0.862) and a combined specificity of 0.808 (95% CI: 0.753–0.853), with a sROC‐generated AUC of 0.822.

The diagnostic AUC of miR‐205, miR‐155 and miR‐19 was also extensively explored, with 13, 8 and 8 evaluations, respectively. The combined AUC for miR‐205 was 0.6802 (95% CI: 0.6404–0.7200; *I* ^2^ = 0.0%), for miR‐155 it was 0.7456 (95% CI: 0.6386–0.8527; *I* ^2^ = 89.7%), and for miR‐19 it was 0.7471 (95% CI: 0.6452–0.8491; *I* ^2^ = 94.8%). The combined sensitivity for miR‐205 was 0.620 (95% CI: 0.560–0.677) and the combined specificity for miR‐205 was 0.719 (95% CI: 0.660–0.771), with a sROC‐generated AUC of 0.646. The combined sensitivity for miR‐155 was 0.856 (95% CI: 0.481–0.974) and the combined specificity for miR‐155 was 0.829 (95% CI: 0.708–0.906), with a sROC‐generated AUC of 0.855. The combined sensitivity for miR‐19 was 0.719 (95% CI: 0.534–0.851) and the combined specificity for miR‐19 was 0.809 (95% CI: 0.714–0.877), with a sROC‐generated AUC of 0.839.

### 3.2 Prognostic Value of MicroRNAs

#### 3.2.1 Overall Findings

**FIGURE 3:**
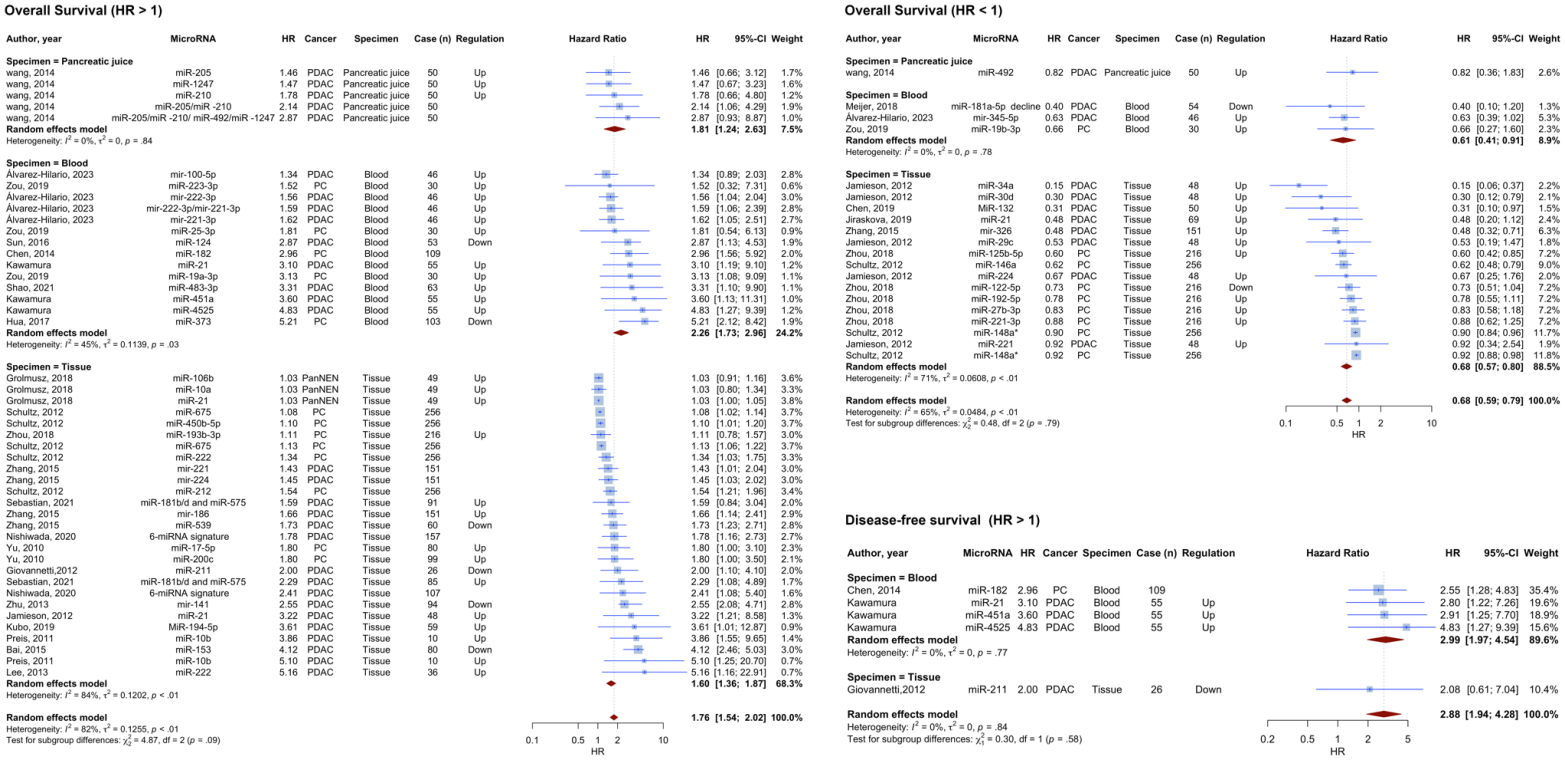
Prognostic value of microRNAs for overall survival (OS) and disease‐free survival (DFS). Overall findings for OS; 46 prognostic analyses HR > 1: combined HR: 1.7613 (95% CI: 1.5394–2.0152; *p* < 0.0001; *I* ^2^ = 81.7%). Subgroup by specimen: tissue: HR 1.5967 (95% CI: 1.3604–1.8741; *I* ^2^ = 83.9%), blood: HR 2.2641 (95% CI: 1.7344–2.9556; *I* ^2^ = 45.5%), pancreatic juice: HR 1.8089 (95% CI: 1.2437–2.6311; *I* ^2^ = 0.0%). Twenty prognostic analyses HR < 1: combined HR: 0.6805 (95% CI: 0.5862–0.7901; *p* < 0.0001; *I* ^2^ = 65.4%). Subgroup by specimen: tissue: HR 0.6760 (95% CI: 0.5697–0.8020; *I* ^2^ = 70%), blood: HR 0.6067 (95% CI: 0.4066–0.9053; *I* ^2^ = 0.0%), pancreatic juice: HR 0.8200 (one evaluation; 95% CI: 0.3637–1.8488). Findings for DFS: 5 prognostic analyses HR > 1: combined HR: 2.8801 (95% CI: 1.9399–4.2759; *p* < 0.0001; *I* ^2^ = 0.0%). Subgroup by specimen: blood: HR 2.9915 (95% CI: 1.9703–4.5419; *I* ^2^ = 0.0%), one tissue evaluation: HR 2.0800 (95% CI: 0.6123–7.0662).

Fourty six prognostic analyses with data on OS reported HR higher than one. The combined HR for these investigations was 1.7613 (95% CI: 1.5394–2.0152, *p* < 0.0001; *I* ^2^ = 81.7%). The evaluations were separated into three subgroups based on the specimen used: blood, tissue, and pancreatic juice. The pooled HR for the tissue specimen subgroup, which included 27 assessments, was 1.5967 (95% CI: 1.3604–1.8741; *I* ^2^ = 83.9%). Involving 14 evaluations, the blood specimen subgroup exhibited a pooled HR of 2.2641 (95% CI: 1.7344–2.9556; *I* ^2^ = 45.5%). The pancreatic juice subgroup with 5 assessments had a pooled HR of 1.8089 (95% CI: 1.2437–2.6311; *I* ^2^ = 0.0%). The test for differences across subgroups was not statistically significant (*p* = 0.0876) (Figure 3). Twenty prognostic evaluations with OS HRs smaller than one resulted in a pooled HR of 0.6805 (95% CI: 0.5862–0.7901, < 0.0001; *I* ^2^ = 65.4%). The pooled HR for the tissue specimen subgroup, which included 16 assessments, was 0.6760 (95% CI: 0.5697–0.8020; *I* ^2^ = 70%). The blood specimen subgroup had three evaluations with a pooled HR of 0.6067 (95% CI: 0.4066–0.9053; *I* ^2^ = 0.0%). The pancreatic juice subgroup had only one investigation, with an HR of 0.8200 (95% CI: 0.3637–1.8488). The test for subgroup differences was not statistically significant (*p* = 0.7851) (Figure 3).

Five prognostic analyses that provided DFS analysis had HRs greater than one. The pooled HR for these investigations was 2.8801 (95% CI: 1.9399–4.2759, *p* < 0.0001; *I* ^2^ = 0.0%). Four studies on tissue specimens yielded a pooled HR of 2.9915 (95% CI: 1.9703–4.5419; *I* ^2^ = 0.0%). Only one study involved tissue samples, with an HR of 2.0800 (95% CI: 0.6123–7.0662) (Figure 3). Only one prognostic analysis that provided DFS analysis had HRs smaller than one with an HR of 0.831 (95% CI: 0.702–0.983). The characteristics of included studies in case of prognosis is provided in Table S3.

#### 3.2.2 Promising MicroRNAs

**FIGURE 4:**
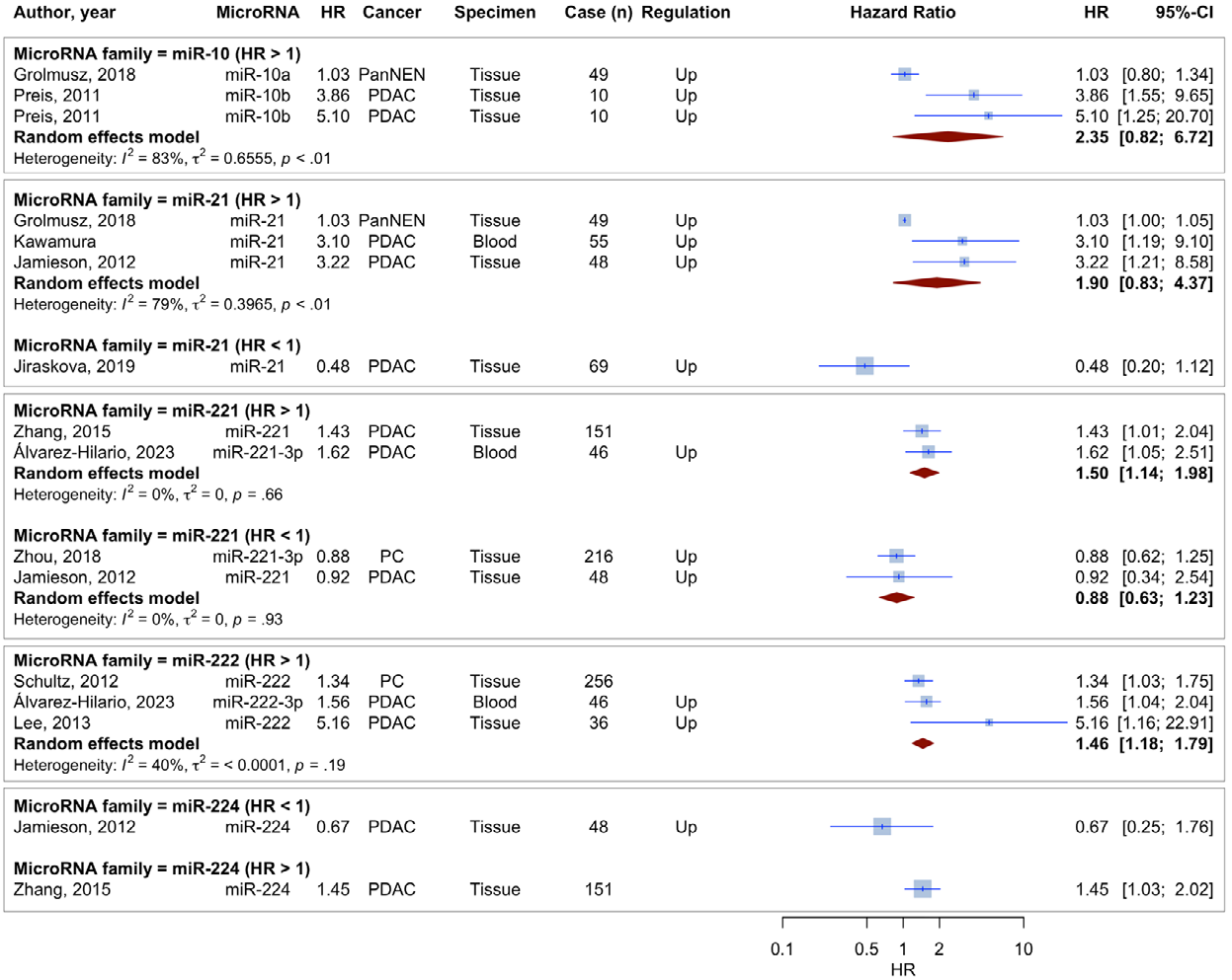
Prognostic value meta‐analysis of the promising microRNAs. Promising microRNAs: MiR‐10: HR > 2 (pooled HR: 2.3538 [0.8241–6.7229]), miR‐21: Pooled HR: 1.9027 [0.8293–4.3652], miR‐221: pooled HR: 1.5022 [1.1424–1.9754].

We performed a meta‐analysis on microRNAs that were the focus of two or more distinct research that reported on their prognostic significance. The findings are concisely displayed in Table 2 and Figure 4. After sorting the findings of the meta‐analyses by the pooled OS HR, it was found that miR‐10 had an HR value of more than 2 (Pooled HR [95% CI] 2.3538 [0.8241–6.7229]). Out of the microRNAs meta‐analysed, miR‐21 (Pooled HR [95% CI] 1.9027 [0.8293–4.3652]), and miR‐221 (Pooled HR [95% CI] 1.5022 [1.1424; 1.9754]) were also promising.

**TABLE 2:** Results of the meta‐analysis summarised for microRNAs with multiple evaluations.

MicroRNA	HR > 1				HR < 1			
	OS evaluations	Pooled OS HR [95% CI]	HR *p*	*I* ^2^	OS evaluations	Pooled OS HR [95% CI]	HR *p*	*I* ^2^
miR‐10	3	2.3538 [0.8241; 6.7229]	0.1099	82.90%				
miR‐21	3	1.9027 [0.8293; 4.3652]	0.1289	79.40%	1	0.4800 [0.2033; 1.1331]	0.094	—
miR‐221	2	1.5022 [1.1424; 1.9754]	0.0036	0.00%	2	0.8843 [0.6344; 1.2325]	0.4679	0.00%
miR‐222	3	1.4554 [1.1850; 1.7876]	0.0003	39.50%				
miR‐224	1	1.4500 [1.0367; 2.0282]	0.03	—	1	0.6700 [0.2525; 1.7777]	0.4212	—

It is important to note that when a certain microRNA has both HR less than one and more than one, it does not necessarily indicate a disagreement between studies. This is because different studies may use different approaches for calculating HR, for example, taking patients with lower expression survival versus patients with higher expression survival, or vice versa.

## 4 Discussion

Patients with pancreatic cancer and their families deal with a huge economic and health burden according to the late imagery identification of the disease [50]. Biopsy tissue remains the gold standard of PC diagnosis; however, it is performed when patients are symptomatic in higher stages. Currently, the only non‐invasive serum factor associated with PC diagnosis is carbohydrate antigen (CA) 19–9 which is not only elevated in chronic pancreatitis, and diabetes mellitus, but also had very low sensitivity and specificity for early diagnosis of PC [37, 51]. Correspondingly, non‐invasive biomarkers such as miRNAs for early‐detection and estimating the prognosis of the pancreatic cancer could significantly alter their morbidity and mortality [52]. In this systematic review and meta‐analysis, we performed 336 evaluations to investigate pooled diagnostic and prognostic values of miRNAs in pancreatic cancer.

### 4.1 Diagnostic Value

Detecting high‐grade dysplasia before the development of malignancy, the time of malignant transformation, and early‐detection of invasive tumour play the most important role in patients' therapeutics and survival [53]. This is the first study that among 290 evaluations (AUC = 0.803), the cumulative analysis of miRNAs of 165 evaluations using blood specimens, 46 studies using tissue specimens, five evaluations using saliva specimens, and 1 study using urine specimens, revealed diagnostic sensitivity, and specificity of 0.732 (95% CI: 0.713–0.750, *p* < 0.001) and 0.748 (95% CI: 0.729–0.766, *p* < 0.001), respectively. Multiple studies assessed circulating miRNAs as diagnostic biomarkers for pancreatic carcinoma compared to healthy controls [51, 54].

A recent multicenter cohort study on tissue and serum specimen of 1273 individuals, evaluated a panel of 27 miRNAs that four serum samples showed promising accuracy with AUC of 0.971 [55]. In our study we performed subgroup analysis of different sample types considering blood, saliva, tissue, pancreatic juice and urine. Urine showed the highest AUC of 0.9, however, it was only mentioned in one trial which highlights the importance of further assessments in order to eliminate potential bias. The source of miRNAs significantly influenced the diagnostic power. Our results demonstrated that tissue, blood, pancreatic juice and saliva had the highest AUC levels, respectively (0.8433, 0.7926, 0.7235, 0.7202). Tisue samples had the most diagnostic validity showing sensitivity of 0.827 and specificity of 0.786. Blood (sensitivity of 0.700 and specificity of 0.745), pancreatic juice (sensitivity of 0.671 and specificity of 0.839), and saliva samples (sensitivity of 0.844 and specificity of 0.457) could be applied, however, single use of each source according to lower rates of accuracy, would be challengeable. Ren and colleagues evaluated the expression of 95 oncogenic miRNAs in PC tissues compared to normal cells, and pancreatic tissues. By revealing eight PC specific miRNAs, authors concluded that cancer tissues express specific miRNAs [56]. Another study also compared tissue samples of cases with pancreatic cancer, chronic pancreatitis and normal pancreas tissue, resulting in overexpression of 21 miRNAs and downregulation of four miRNAs specific to cancer tissues [57]. Another study also proposed a combination of increased plasma levels of miR‐125a‐3p, miR‐92a‐2‐5p and miR‐4530 could identify patients with PC in early stages. Additionally, they highlighted the importance of convenient sampling method in screening test, prioritising serum and blood concentrations of miRNAs although less sensitive or specific (7). A meta‐analysis on diagnostic accuracy of blood‐derived miRNAs for PC, Li et al. indicted a pooled sensitivity of 0.88, pooled specificity of 0.83, and AUC of 0.90 [54]. Not only the study had limited number of patients and controls, but also lacked proposing a panel and miR‐21, as a general oncogenic miRNA, was the only single miRNA evaluated.

Our findings on 165 evaluations showed a sensitivity of 0.700 (95% CI: 0.679–0.720), a cumulative specificity of 0.745 (95% CI: 0.724–0.765), and sROC‐generated AUC was 0.782. Well‐known oncogenic miRNAs showed the same alterations among tissue samples of PC patients as well. Tumour suppressor miRNAs such as miR‐15a, miR‐16‐1, miR‐126 and miR‐200, alongside oncogenic miRNAs such as miR‐21, miR‐221 and miR‐155 were down and upregulated, respectively [58]. Our findings underlined eight miRNAs with diagnostic AUC higher than 0.8 including miR‐1290, miR‐93, miR‐25, miR‐451, miR‐20, miR‐21, miR‐223 and miR‐122, respectively. To furtherly explore the role of a single miRNA in PC early‐diagnosis, miR‐21, miR‐205, miR‐155 and miR‐19 were chosen. Li et al. pointed that the diagnostic value of miR‐21 was higher than panels of miRNAs, suggesting a promising diagnostic marker in PC [59]. This meta‐analysis demonstrated that miR‐21 sensitivity and specificity had a combined sensitivity of 0.774 (95% CI: 0.653–0.862) and a combined specificity of 0.808 (95% CI: 0.753–0.853), with a sROC‐generated AUC of 0.822. Studies, previously approved the considerable role of miR‐21, subjected to transcriptional regulation, in migration and invasion, apoptosis and cell cycle mediating oncogenic effects [60, 61]. Among patients suffering gastric cancer, colorectal cancer, glioma, breast cancer, lung cancer, prostate cancer and particularly other gastrointestinal malignancies, miR‐21 was significantly upregulated with high diagnostic sensitivity and specificity [62, 63, 64]. miR‐21 targets phosphatase and tensin homologue (PTEN), programed cell death 4 (PDCD4), tropomyosin 1 (TMP1), and tissue inhibitor of metalloproteinases 3 (TIMP3) genes in pancreatic cancer resulting in increased proliferation, invasion and chemoresistance [65, 66, 67]. The PIK3/AKT pathway also inhibit cell apoptosis. PTEN gene which is a suppressor of PI3K‐AKT–mTOR signalling, is the target of miR‐21, miR‐221 and miR‐181a, which are responsible for cell cycle arrest, cell proliferation and migration of PC cells, respectively [61, 68, 69, 70]. Furthermore, Zhao et al., demonstrated a positive feedback between miR‐21 and epidermal growth factor (EGF) signalling pathway in PC; miR‐21 is promoted by EGF, simultaneously inhibiting EGF inhibitors [71].

Results of this study proposed that miR‐320 with an AUC of 0.965 showed the highest diagnostic validity. This novel biomarker has demonstrated highly sensitive and specific diagnostic value in retinoblastoma, colorectal and breast cancer [72]. Similarly, in this study we approved that this miRNA could be a better single detector compared to different panels. To date miR‐39 signature via a machine learning model displayed an accuracy of 0.93 and AUC of 0.98 [73]. Further, the diagnostic value of miR‐21 in adenocarcinoma of pancreas exhibited sensitivity, specificity, and AUC of 0.90, 0.72 and 0.91, respectively [54]. There are also panels with AUC above 0.9. Shams et al. through bioinformatics analysis reported miR‐125a‐3p, miR‐5100 and miR‐642b‐3p with AUC of 0.95, sensitivity of 0.98, and specificity of 0.97 demonstrating an outstanding diagnostic value [74]. Consistently, based on tissue and serum sample of 1273 individuals, a very recent study reported a panel of four miRNAs including miR‐132‐3p, miR‐30c‐5p, miR‐24‐3p and miR‐23a‐3p with a highly promising accuracy with AUC of 0.971 [55]. In addition, a panel of 2′‐O‐methylated (2′OMe) miRNAs (miR‐28‐3p, miR‐143‐3p, miR‐151a‐3p) also showed AUC of 0.928 when comparing pancreatic ductal adenocarcinoma and healthy participants [75].

We also assessed miR‐205, miR‐155 and miR‐19 extensively. The combined sensitivity and specificity for miR‐205 were lower than 0.7, however, Zhuang and colleagues emphasised that lower concentrations of this miRNA lead to further PC invasion [76]. miR‐155 revealed sensitivity and specificity of 0.856, and 0.829, respectively, however, it plays a significant diagnostic role among cancers with the highest accuracy for leukaemia [77, 78]. This biomarker, targets tumour protein 53‐induced nuclear protein 1 (TP53INP1) gene, and the upregulation led to augmented proliferation and tumorigenesis in PC [79, 80, 81]. Moreover, the oncogenic miR‐155 is associated to JAK/STAT pathway, downregulating suppressor gene SOCS1, resulting in invasion and migration fo pancreatic ductal adenocarcinoma cell [82]. Similar to miR‐155, miR‐19 has promising diagnostic accuracy for PC, while its oncogenic role among non‐small cell lung cancer, and cervical cancer was confirmed [83, 84]. This broad implementation highlights the importance of large‐scale validations before applying in PC or any other type of cancer. Volinia et al. applying microarray on 363 tissue samples of different tumour types including pancreas, lung, breast, stomach, prostate and colon reported a cluster containing 137 miRNAs could be a tissue indicator [85]. Similarly, a long‐term study on various types of cancers showed that upregulation of miR‐216 and miR‐217, in addition to downregulation of miR‐133a are specifically determined to pancreatic cells [79]. However, miRNA‐216a, as the third downregulated miRNA in pancreatic ductal adenocarcinoma, is involved in the Janus kinase/signal transducers and activators of transcription (JAK/STAT) pathway stimulating cell proliferation, differentiation and migration. JAK2 mRNA in directly inhibited by miRNA‐216a resulting in a reduction of tumour volume [68, 86]. We recommend designing panels containing both PC specific and broad promising miRNAs with high specificity and sensitivity, for early specific and sensitive diagnosis.

### 4.2 Prognostic Value

Estimating the prognosis of PC, affecting their quality of life, particularly when the tumour is irresectable. Evaluating the probable course of the disease prior to treatment could significantly alter the patients' situation. Yan et al. evaluated serum miRNA signature of PC patients [87]. Authors divided 100 PC samples into 21 operable and 79 inoperable, and listed 432 miRNAs showing operability of tumour showing sensitivity, specificity, and accuracy of 0.857, 0.848, and 0.850, respectively. Therefore, prognostic non‐invasive biomarkers such as miRNAs. We performed 46 prognostic analyses with HR higher than one (1.7613, 95% CI: 1.5394–2.0152). The HRs regarding the sample type were as follows; the blood specimen (2.2641), tissue specimen (1.5967), and pancreatic juice (1.8089), with no significant difference across subgroups. Twenty prognostic evaluations with OS HRs smaller than one showed a pooled HR of 0.6805 (95% CI: 0.5862–0.7901). The subgroup analysis revealed no difference among different sample sources.

According to our results, the three most promising miRNAs demonstrating the highest HR were miR‐10, with an HR value more than 2, miR‐21 with a pooled HR of 1.9027, and miR‐221 showing pooled HR of 1.5022. miR‐221 cause proliferation of tumour by targeting cyclin‐dependent kinase inhibitor 1B (CDKN1B (p27)), upregulated modulator of apoptosis (PUMA (p53)), and PTEN genes [88, 89, 90, 91]. Nakata and colleagues through a microarray analysis, reported that miR‐10b expression is strongly associated with a lesser overall survival, as the higher the miR‐10b, the more invasive the PC [92]. In patients with pancreatic ductal adenocarcinoma, reduced concentrations of miR‐10b were correlated with better response to multimodality neoadjuvant therapy, surgery, late metastasis and augmented survival [93]. A meta‐analysis conducted by Zhou et al., showed that miR‐21 is significantly related with poor overall survival of gastrointestinal tumours (HR = 1.68), pancreatic cancer (HR = 2.53), lung cancer (HR = 1.59), breast cancer (HR = 2.55), and liver cancer (HR = 1.93) [94]. Moreover, poor DFS was related with miR‐21 elevation in pancreatic cancer (HR of 2.87) [95]. This miRNA also played an important predictive role in sensitivity to gemcitabine chemotherapy regimen in patients with advanced stages of PC (III and IV) [96]. High level of miR‐21 resulted in apoptosis resistance after therapy, suggesting a promising therapeutic target when inhibited in combination with gemcitabine could develop further angiogenesis, provide higher concentrations of medications, and induce tumour regression [33]. There still lack a specific prognostic miRNA in PC. As mentioned, molecules such as miR‐21 is involved in varied types of cancers, it would instead express a carcinogenic process in any tissue rather than PC prognosis. A combination of these absolute biomarkers with specific miRNAs for each condition, which require further investigations in PC patients.

This meta‐analysis confirms the diagnostic and prognostic role of miRNAs in PC. We investigated the diagnostic and prognostic value of miRNAs in PC according to their sample source. We provided a list of the most accurate miRNAs for both diagnosis and prognosis of the disease. miRNAs are activated in a row, thus, there are up and down stream molecules. Detecting the key miRNA through further large‐scale prospective studies to unravel the molecular mechanism of these miRNA signature in PC, help further establishment of not only diagnostic and prognostic panels, but also improve miRNA targeted therapy. More exclusively, Zhan et al. suggested that upregulation of miR‐455‐3p as a tumour suppressor, promoted the apoptosis of cancerous cells by impacting the expression of Bcl‐2, and Bax apoptotic proteins via Wnt/catenin signalling pathway [97]. Furthermore, miR‐373‐3p by inhibiting the regulation of *Cycin D2*, improve gemcitabine chemosensitivity, as well as reducing gemcitabine‐resistant PC cells [98]. miRNA antagonists, single‐stranded antisense oligodeoxynucleotides (ASO), are promising targeted therapeutics that hinder carcinogenic miRNAs [99]. The overexpression of suppressors and downregulation of oncogenic miRNAs, would lead to tumour inhibition.

However, this study has limitation. The relatively small sample size of patients and controls might alter the results of the meta‐analysis, and we had to exclude some studies due to insufficient data. There were considerable differences among studies, such as the time of sample storage, miRNA extraction and detection methods, which could bias the results. Additionally, various methods for quantitative assessments regarding the influence of different RNA processing methods on miRNA transcript levels could affect the outcomes. Ethnicity, sex, age and cancer stage are factors that significantly affect miRNA concentrations, but due to inadequate data, we could not perform subgroup analysis on each factor to provide the most accurate miRNA panels according to the stage of the disease and demographics. There was lack of sufficient miRNA panel assessment studies, and we could not include panels in the meta‐analysis. Most of literature, focused on known miRNAs such as miR‐21, while there are also other less marked such biomarkers that further evaluations on them could significantly alter miRNA role in diagnosis and prognosis of PC. The majority of the studies included in the diagnostic section of our systematic review and meta‐analysis were conducted in China, highlighting that the largest number of articles originated from this country. Additionally, studies from the following countries were included: Japan, the United States, Germany, Romania, Iran, Egypt, the Czech Republic, India, Brazil, Mexico, Spain, Korea, Poland, and data from 23 research centers across 10 European countries. For the prognostic section, the majority of studies were conducted in China, Additionally, studies from the following countries were included: The United States, the Netherlands, Japan, Italy, the United Kingdom, the Czech Republic, Mexico, Hungary, Germany, and Denmark. Due to inadequate data, we could not perform subgroup analysis on each, and provide a least of the most accurate miRNA panels according to stage of the disease and demographics. Further large‐scale prospective studies using different emerging technologies are necessary to validate the role of miRNAs in PC, as well as constructing proper diagnostic and prognostic panels with the highest accuracy.

This study included investigations from different regions, using various assays and involving diverse patient populations, introducing significant heterogeneity. This heterogeneity can affect the comparability and overall interpretation of the study results. To better understand how these factors influence the results, it is crucial to explore these sources of heterogeneity in greater depth. Meta‐regression or subgroup analysis could be employed to assess the impact of regional differences, assay variations and patient demographic factors on the study outcomes.

Future studies should aim to include larger sample sizes to enhance the robustness of the conclusions. Employing emerging detection technologies, such as single‐cell sequencing, to validate and refine the role of miRNAs in pancreatic cancer diagnosis and prognosis will be vital. These approaches will help to establish more reliable diagnostic and prognostic panels and improve miRNA‐targeted therapy.

## Author Contributions

**Fatemeh Hasani:** conceptualization (equal), data curation (equal), formal analysis (equal), investigation (equal), methodology (equal), project administration (equal), software (equal), writing – original draft (equal), writing – review and editing (equal). **Mahdi Masrour:** conceptualization (equal), data curation (equal), formal analysis (equal), investigation (equal), methodology (equal), project administration (equal), software (equal), supervision (equal), writing – original draft (equal), writing – review and editing (equal). **Sina Khamaki:** conceptualization (equal), data curation (equal), investigation (equal), methodology (equal), writing – original draft (equal). **Kimia Jazi:** conceptualization (equal), data curation (equal), resources (equal), software (equal), writing – original draft (equal). **Saba Hosseini:** conceptualization (equal), validation (equal), writing – original draft (equal). **Hadiseh Heidarpour:** validation (equal), writing – original draft (equal). **Mehrad Namazee:** software (equal), validation (equal), writing – original draft (equal).

## Ethics Statement

The authors have nothing to report.

## Conflicts of Interest

The authors declare no conflicts of interest.

## Supporting information


**Data S1**.

## Data Availability

All data used for this manuscripts is included in the main or Supporting Information S1.
