# Supplementary material for: Diagnostic and Prognostic Accuracy of MiRNAs in Pancreatic Cancer: A Systematic Review and Meta‐Analysis
Source: J Cell Mol Med. 2025 Jan 24;29(2):e70337. doi: 10.1111/jcmm.70337 (PMC11761000; doi:10.1111/jcmm.70337)

**Supplementary Information**

**Supplementary Table 1.** The complete search algorithms for PubMed, Web of Sciences, and Scopus library are as follows.

| Database | Syntax | Results |
| --- | --- | --- |
| PubMed | (((((((((((((Pancreatic Neoplasm[Title/Abstract]) OR (Pancreatic Cancer[Title/Abstract])) OR (Pancreatic Cancers[Title/Abstract])) OR (Pancreatic Neoplasms[Title/Abstract])) OR (Pancreas Neoplasms[Title/Abstract])) OR (Pancreas Neoplasm[Title/Abstract])) OR (Pancreas Cancers[Title/Abstract])) OR (Pancreas Cancer[Title/Abstract])) OR (Cancer of the Pancreas[Title/Abstract])) OR (Cancer of Pancreas[Title/Abstract])) OR (Pancreatic carcinoma[Title/Abstract])) OR (Pancreatic adenocarcinoma[Title/Abstract])) AND ((((((((Diagnos*[Title/Abstract]) OR (Diagnosis[Title/Abstract])) OR (Diagnoses[Title/Abstract])) OR (Postmortem Diagnosis[Title/Abstract])) OR (Postmortem Diagnoses[Title/Abstract])) OR (Antemortem Diagnosis[Title/Abstract])) OR (Antemortem Diagnoses[Title/Abstract]) ) OR (prog*[Title/Abstract]))) AND (((((((((((microRNA*[Title/Abstract]) OR (miRNA*[Title/Abstract])) OR (micro RNA[Title/Abstract])) OR (Primary microRNA[Title/Abstract])) OR (Primary miRNA[Title/Abstract])) OR (pri-miRNA[Title/Abstract])) OR (pri miRNA[Title/Abstract])) OR (stRNA[Title/Abstract])) OR (Small Temporal RNA[Title/Abstract])) OR (pre-miRNA[Title/Abstract])) OR (pre miRNA[Title/Abstract])) | 1,263 |
| Web of Science | TS=(“Pancreatic Neoplasm” OR “Pancreatic Cancer” OR “Pancreatic Cancers” OR “Pancreatic Neoplasms” OR “Pancreas Neoplasms” OR “Pancreas Neoplasm” OR “Pancreas Cancers” OR “Pancreas Cancer” OR “Cancer of the Pancreas” OR “Cancer of Pancreas” OR “Pancreatic carcinoma” OR “Pancreatic adenocarcinoma”) AND TS=(“Diagnos*” OR “Diagnosis” OR “Diagnoses” OR “Postmortem Diagnosis” OR “Postmortem Diagnoses” OR “Antemortem Diagnosis” OR “Antemortem Diagnoses” OR "prog*”) AND TS=(”microRNA” OR “miRNA*” OR “micro RNA” OR “Primary microRNA” OR “Primary miRNA” OR “pri-miRNA” OR “pri miRNA” OR “stRNA” OR “Small Temporal RNA” OR “pre-miRNA” OR “pre miRNA”) | 1,837 |
| Scopus | TITLE-ABS-KEY(“Pancreatic Neoplasm” OR “Pancreatic Cancer” OR “Pancreatic Cancers” OR “Pancreatic Neoplasms” OR “Pancreas Neoplasms” OR “Pancreas Neoplasm” OR “Pancreas Cancers” OR “Pancreas Cancer” OR “Cancer of the Pancreas” OR “Cancer of Pancreas” OR “Pancreatic carcinoma” OR “Pancreatic adenocarcinoma”) AND TITLE-ABS-KEY(“Diagnos*” OR “Diagnosis” OR “Diagnoses” OR “Postmortem Diagnosis” OR “Postmortem Diagnoses” OR “Antemortem Diagnosis” OR “Antemortem Diagnoses” OR "prog*”) AND TITLE-ABS-KEY(”microRNA” OR “miRNA*” OR “micro RNA” OR “Primary microRNA” OR “Primary miRNA” OR “pri-miRNA” OR “pri miRNA” OR “stRNA” OR “Small Temporal RNA” OR “pre-miRNA” OR “pre miRNA”) | 3,314 |
| Total = 6,414  After deleting duplicated files = 4,183 | |  |

**Supplementary Table 2.** The characteristics of included studies in case of diagnosis

| **ID** | **Author, year** | **country** | **Study type** | **Cancer type** | **PC def** | **HC def** | **Age Case** | **Age control** | **sample case** | **sample control** | **case N** | **control N.** | **miRNA** | **down/up** | **Sensitivity** | **Specificity** | **AUC** | **upper ci** | **lower ci** | **p-value** | **NPV** | **PPV** |
| --- | --- | --- | --- | --- | --- | --- | --- | --- | --- | --- | --- | --- | --- | --- | --- | --- | --- | --- | --- | --- | --- | --- |
| 1 | Zou, 2019 | China | cohort | PC | histopathological confirmed PC patients | healthy donors who took routine health checkup | ≤60 : 11 (36.7%) | ≤60: 13 (43.3%) | serum | serum | 30 | 30 | miR‐25‐3p | up | 66.7 | 80.4 | 0.726 | 0.792 | 0.659 | _ | _ | _ |
|  |  |  |  |  |  |  |  |  |  |  |  |  | let‐7b‐5p | up | 79.8 | 59.8 | 0.703 | 0.771 | 0.636 | _ | _ | _ |
|  |  |  |  |  |  |  |  |  |  |  |  |  | miR‐192‐5p | up | 77.5 | 57 | 0.684 | 0.754 | 0.615 | _ | _ | _ |
|  |  |  |  |  |  |  |  |  |  |  |  |  | miR‐19a‐3p | up | 71.3 | 78.5 | 0.771 | 0.831 | 0.712 | _ | _ | _ |
|  |  |  |  |  |  |  |  |  |  |  |  |  | miR‐19b‐3p | up | 65.1 | 81.3 | 0.788 | 0.846 | 0.729 | _ | _ | _ |
| 2 | Wang, 2019 | China | Case-control | PC | Diagnosed through pathological examinations. No cases had underwent chemotherapy or radiotherapy ahead of surgical opera | healthy persons who visited the hospital for physical examination. | 58.16 ± 12.31 | 60.13 ± 11.56 | serum | serum | 110 | 64 | microRNA‐133a | down | 90.6 | 87.2 | 0.893 | _ | _ | _ | _ | _ |
| 3 | Ishige. 2020 | Japan | Case-control | PC:  PDAC(n=36),  IPMN   with high-grade  dysplasia(n=2),   IPMN-associated PC(n=2)  and ,anaplastic carcinoma( n=1) | Pancreatic cancer | Healthy control | 68.1 (48–89) | 62.5 (51–76) | saliva | saliva | 41 | 30 | MIR1246 | did not differ | 91 | 26.7 | 0.48 | _ | _ | _ | _ | _ |
|  |  |  |  |  |  |  |  |  | serum | serum |  |  | MIR1246 | up | 92.3 | 73.3 | 0.87 | _ | _ | _ | _ | _ |
|  |  |  |  |  |  |  |  |  | Urine | Urine |  |  | MIR1246 | up | 90.2 | 83.3 | 0.9 | _ | _ | _ | _ | _ |
| 4 | Nakamura, 2019 | Japan | Case-control | PDAC | diagnosis of PDAC was made by pathology or cytology | Chronic pancratitis, diagnosis of CP was made by pathology or clinical criteria  based on imaging results | 71 (47–79) | 59.5 (38–79) | serum | serum | 27 | 8 | miR-21 | up | 81 | 88 | 0.9 | _ | _ | _ | 58 | 96 |
|  |  |  |  |  |  |  |  |  | Pancreatic juice | Pancreatic juice |  |  | miR-21 | no difference | _ | _ | 0.71 | _ | _ | _ | _ | _ |
|  |  |  |  |  |  |  |  |  | serum | serum |  |  | miR-155 | up | 89 | 88 | 0.89 | _ | _ | _ | _ | _ |
|  |  |  |  |  |  |  |  |  | Pancreatic juice | Pancreatic juice |  |  | miR-155 | no difference | _ | _ | 0.56 | _ | _ | _ | 70 | 96 |
| 5 | Dorbe, 2023 | Romania | Cohort | PDAC | _ | Non-tumoral pancreatic tissues from individuals who underwent pancreatic surgical resection for differ ent reasons: surgical resection for a PDAC (n = 8), a neuroendocrine tumor (n = 4), an intraductal papillary mucinous neoplasm (n = 2), an adenosquamous carcinoma (n = 1), and metastasis of colorectal carcinoma (n = 1). | 61.23 ± 8.91 | 56.18 ± 12.29 | Tissue | Tissue | 30 | 16 | mir-20b-5p | up | 92.9 | 75 | 0.768 | _ | _ | 0.013 | _ | _ |
|  |  |  |  |  |  |  |  |  | Tissue | Tissue | 30 | 16 | mir-15b-5p | up | 92.9 | 83.1 | 0.714 | _ | _ | 0.046 | _ | _ |
| 6 | Chen, 2014 | China | Case-control | PC | The diagnosis of PCa was based on pathological evaluation of surgical specimens or biopsies | Chronic pancratitis(n=38), healthy volunteers were recruited as healthy controls (n=50) | >50 : 41 | _ | Serum | Serum | 109 | 88 | mir-182 | up | 64.1 | 82.6 | 0.775 | 0.856 | 0.721 | _ | _ | _ |
| 7 | Chen, 2022 | China | Case-control | PC | _ | Pancreatic benign disease | 62±9.15 | 59±10.17 | Serum | Serum | 191 | 95 | miR-451a | up | 71.2 | 89.47 | 0.855 | 0.899 | 0.812 | _ | _ | _ |
|  |  |  |  |  |  | HC, individuals seeking a routine health checkup at this hospital, who showed no evidence of cancers or other diseases | 62±9.15 | 57±11.32 | Serum | Serum | 191 | 90 | miR-451a | up | 80.1 | 86.67 | 0.896 | 0.932 | 0.86 | _ | _ | _ |
| 8 | Deng, 2016 | China | Case-control | PC | PC diagnosis confirmed by pathological examination; b) no acute infection; c) no jaundice; and d) alanine aminotransferase (ALT) and aspartate aminotransferase (AST) levels less than 2.5 times the upper limit of normal. | Normal control (n=600), other disease (n=160) | 62±10 | NC: 49±6, other dis: 56±13 | Serum | Serum | 303 | 760 | miR-25 | up | 75.58 | 93.03 | 0.915 | 0.937 | 0.893 | _ | _ | _ |
| 9 | Seyed Salehi, 2022 | Iran | cohort | PC | Histopathological confirmed PC patients. | Normal control (noncancerous donors) | _ | _ | Serum | Serum | 77 | 65 | miR-92a-2-5p | up | 80.1 | 68.4 | 0.73 | 0.831 | 0.686 | <0.0001 | _ | _ |
|  |  |  |  |  |  |  |  |  |  |  |  |  | miR-125a-3p | up | 86.8 | 68.8 | 0.85 | 0.921 | 0.746 | <0.0001 | _ | _ |
|  |  |  |  |  |  |  |  |  |  |  |  |  | miR-4530 | up | 81.5 | 64 | 0.76 | 0.854 | 0.615 | <0.0001 | _ | _ |
| 10 | Duell 2017 | Twenty three research centers in 10 European countries | cohort | PDAC | PDAC(≤12 yr. follow up) | Control participants were selected randomly among EPIC participants who were alive and free of cancer (except non-melanoma skin cancer).  At the time of diagnosis of each index case and matched to each case based on sex, study center or country, age at blood collection (+/- 3 years), date of blood collection (+/- 3 months), time of blood collection (+/- 2 hours), fasting status (<3 hours, 3-6 hours, >6 hours after last meal), and among women, use of hormones (OC, HRT). | _ | _ | Serum | Serum | 225 | 225 | mir-10a | up | _ | _ | 0.66 | 0.71 | 0.61 | _ | _ | _ |
|  |  |  |  |  |  |  |  |  |  |  |  |  | miR-10b | up | _ | _ | 0.68 | 0.73 | 0.63 | _ | _ | _ |
|  |  |  |  |  |  |  |  |  |  |  |  |  | miR-21-3p | no difference | _ | _ | 0.63 | 0.68 | 0.58 | _ | _ | _ |
|  |  |  |  |  |  |  |  |  |  |  |  |  | miR-21-5p | up | _ | _ | 0.64 | 0.69 | 0.59 | _ | _ | _ |
|  |  |  |  |  |  |  |  |  |  |  |  |  | miR-30c | up | _ | _ | 0.71 | 0.76 | 0.66 | _ | _ | _ |
|  |  |  |  |  |  |  |  |  |  |  |  |  | miR-106b | no difference | _ | _ | 0.64 | 0.69 | 0.59 | _ | _ | _ |
|  |  |  |  |  |  |  |  |  |  |  |  |  | miR-155 | up(not significant) | _ | _ | 0.64 | 0.69 | 0.59 | _ | _ | _ |
|  |  |  |  |  |  |  |  |  |  |  |  |  | miR-212 | up | _ | _ | 0.64 | 0.69 | 0.59 | _ | _ | _ |
|  |  |  |  |  | PDAC(≤8 yr. follow up) |  |  |  |  |  | 53 | 53 | mir-10a | no difference | _ | _ | 0.72 | 0.79 | 0.66 | _ | _ | _ |
|  |  |  |  |  |  |  |  |  |  |  |  |  | miR-10b | up | _ | _ | 0.73 | 0.8 | 0.67 | _ | _ | _ |
|  |  |  |  |  |  |  |  |  |  |  |  |  | miR-21-3p | no difference | _ | _ | 0.71 | 0.77 | 0.64 | _ | _ | _ |
|  |  |  |  |  |  |  |  |  |  |  |  |  | miR-21-5p | up | _ | _ | 0.72 | 0.78 | 0.65 | _ | _ | _ |
|  |  |  |  |  |  |  |  |  |  |  |  |  | miR-30c | up | _ | _ | 0.76 | 0.82 | 0.7 | _ | _ | _ |
|  |  |  |  |  |  |  |  |  |  |  |  |  | miR-106b | no difference | _ | _ | 0.7 | 0.76 | 0.63 | _ | _ | _ |
|  |  |  |  |  |  |  |  |  |  |  |  |  | miR-155 | no difference | _ | _ | 0.71 | 0.77 | 0.64 | _ | _ | _ |
|  |  |  |  |  |  |  |  |  |  |  |  |  | miR-212 | no difference | _ | _ | 0.7 | 0.77 | 0.63 | _ | _ | _ |
|  |  |  |  |  | PDAC(≤5 yr. follow up) |  |  |  |  |  | 29 | 29 | mir-10a | no difference | _ | _ | 0.75 | 0.83 | 0.66 | _ | _ | _ |
|  |  |  |  |  |  |  |  |  |  |  |  |  | miR-10b | up | _ | _ | 0.76 | 0.84 | 0.68 | _ | _ | _ |
|  |  |  |  |  |  |  |  |  |  |  |  |  | miR-21-3p | no difference | _ | _ | 0.74 | 0.82 | 0.65 | _ | _ | _ |
|  |  |  |  |  |  |  |  |  |  |  |  |  | miR-21-5p | up | _ | _ | 0.74 | 0.87 | 0.71 | _ | _ | _ |
|  |  |  |  |  |  |  |  |  |  |  |  |  | miR-30c | up | _ | _ | 0.79 | 0.85 | 0.69 | _ | _ | _ |
|  |  |  |  |  |  |  |  |  |  |  |  |  | miR-106b | no difference | _ | _ | 0.74 | 0.83 | 0.65 | _ | _ | _ |
|  |  |  |  |  |  |  |  |  |  |  |  |  | miR-155 | no difference | _ | _ | 0.74 | 0.83 | 0.66 | _ | _ | _ |
|  |  |  |  |  |  |  |  |  |  |  |  |  | miR-212 | no difference | _ | _ | 0.73 | 0.82 | 0.65 | _ | _ | _ |
| 11 | Goto, 2018 | Japan | cohort | IPMN(branched-duct type  (BD)-IPMN) | _ | HC, without malignant or neoplastic lesions were registered in the control group | 73.8 ± 7.8 | 57.5 ± 15.3 | Serum | Serum | 29 | 22 | mir-191 | Up | 64.3 | 79 | 0.741 | 0.884 | 0.597 | _ | _ | _ |
|  |  |  |  |  |  |  |  |  |  |  |  |  | mir-21 | Up | 75.9 | 81 | 0.741 | 0.882 | 0.599 | _ | _ | _ |
|  |  |  |  |  |  |  |  |  |  |  |  |  | mir-451a | Up | 62.1 | 85.7 | 0.742 | 0.879 | 0.606 | _ | _ | _ |
|  |  |  |  | early stage PC (stage I or IIa) |  |  | 64.0 ± 10.1 | 57.5 ± 15.3 |  |  | 9 | 22 | mir-191 | Up | 66.7 | 84.2 | 0.754 | 0.966 | 0.543 | _ | _ | _ |
|  |  |  |  |  |  |  |  |  |  |  |  |  | mir-21 | Up | 66.7 | 81 | 0.741 | 0.937 | 0.545 | _ | _ | _ |
|  |  |  |  |  |  |  |  |  |  |  |  |  | mir-451a | Up | 66.7 | 85.7 | 0.735 | 0.952 | 0.519 | _ | _ | _ |
|  |  |  |  | advanced-stage PC (stage ≥IIb) |  |  | 64.0 ± 10.1 | 57.5 ± 15.3 |  |  | 23 | 22 | mir-191 | Up | 78.6 | 79 | 0.801 | 0.944 | 0.658 | _ | _ | _ |
|  |  |  |  |  |  |  |  |  |  |  |  |  | mir-21 | Up | 86.4 | 81 | 0.862 | 0.972 | 0.761 | _ | _ | _ |
|  |  |  |  |  |  |  |  |  |  |  |  |  | mir-451a | Up | 69.6 | 81 | 0.768 | 0.911 | 0.626 | _ | _ | _ |
|  |  |  |  | PC |  |  | 64.0 ± 10.1 | 57.5 ± 15.3 |  |  | 32 | 22 | mir-191 | Up | 71.9 | 84.2 | 0.788 | 0.918 | 0.657 | _ | _ | _ |
|  |  |  |  |  |  |  |  |  |  |  |  |  | mir-21 | Up | 80.7 | 81 | 0.826 | 0.938 | 0.715 | _ | _ | _ |
|  |  |  |  |  |  |  |  |  |  |  |  |  | mir-451a | Up | 65.6 | 85.7 | 0.759 | 0.888 | 0.63 | _ | _ | _ |
| 12 | Hua, 2017 | China | Case-control | PC | No prior pancreatic cancer surgery, or other concomitant cancers, or a history of HIV infection | HC, healthy volunteers, who were proved not to have any pancreatic disease or other cancerous disease | > 65: 68 | _ | Serum | Serum | 103 | 50 | miR-373 | down | 80.6 | 84.3 | 0.852 | _ | _ | _ | _ | _ |
| 13 | Hussein, 2016 | Egypt | Case-control | PDAC | No prior pancreatic cancer surgery, or other concomitant cancers, or a history of HIV infection | HC, healthy participants with no family history of pancreatic cancer | 57.43 ± 10.82 | 40.93 ± 9.26 | Serum | Serum | 35 | 15 | MiR-22-3p | Up | 97.14 | 93.33 | 0.943 | _ | _ | _ | _ | _ |
|  |  |  |  |  |  |  |  |  |  |  |  |  | MiR-642b-3p | Up | 100 | 100 | 1 | _ | _ | _ | _ | _ |
|  |  |  |  |  |  |  |  |  |  |  |  |  | MiR-885-5p | Up | 100 | 100 | 1 | _ | _ | _ | _ | _ |
| 14 | Liu, 2016 | China | Case-control | PDAC | PDAC | adjacent normal pancreatic tissues | 58.75±2.210 vs. 59.24±1.348 | _ | Tissue | Tissue | 57 | 25 | miR-23b-3p | down | 68.4 | 60 | 0.7162 | 0.8515 | 0.5809 | 0.0042 | _ | _ |
| 15 | Yu, 2021 | China | Case-control | PC | patients who did not receive surgical treatment before the examination; all patients confirmed by clinical examination and pathological examination; and complete clinical medical records. Exclusion criteria: patients with cirrhosis; patients with coagulation dys function or liver cancer metastatic; patients with car diovascular and visceral diseases; complicated with other malignant tumors; confused consciousness or accom panied by mental illness; and patients with MRI contraindication | HC | 46.77 ± 10.65 | 48.13 ± 10.71 | serum | serum | 160 | 100 | miR-21 | down | 58.13 | 79 | Accuracy: 66.15% | _ | _ | _ | _ | _ |
| 16 | Karasek, 2018 | Czech Republic | cohort | PDAC | patients diagnosed for PDAC who underwent surgical resection | HC, cancer-free controls with no history of any type of cancer. | >65: 12 | 64 (45-79) | serum | serum | 25 | 24 | miR-21-5p | up | _ | _ | 0.99 | | _ | <0.001 | _ | _ |
|  |  |  |  |  |  |  |  |  |  |  |  |  | miR-375 | up | _ | _ | 0.73 | | _ | <0.05 | _ | _ |
|  |  |  |  |  |  |  |  |  |  |  |  |  | miR-155 | up | _ | _ | 0.86 | | _ | <0.001 | _ | _ |
|  |  |  |  |  |  |  |  |  |  |  |  |  | miR-17-5p | up | _ | _ | 0.87 | | _ | <0.001 | _ | _ |
|  |  |  |  |  |  |  |  |  |  |  |  |  | miR-126-5p | up | _ | _ | 0.85 | | _ | <0.001 | _ | _ |
|  |  |  |  |  |  |  |  |  |  |  |  |  | miR-1290 | up | _ | _ | 0.78 | | _ | <0.05 | _ | _ |
| 17 | Ahmad Khan, 2021 | India | Case-control | PDAC | PDAC was diagnosed on the basis of radiological or histopathological examinations | HC, normal pancreatic tissue specimens from autopsy cases | 55.65±11.67 | _ | serum | serum | 50 | 25 | miR-215-5p | up | 72 | 72 | 0.832 | | 0.904 | 0.721 | _ | _ |
|  |  |  |  |  |  |  |  |  |  |  |  |  | miR-122-5p | up | 98 | 96 | 0.988 | | 0.999 | 0.927 | _ | _ |
|  |  |  |  |  |  |  |  |  |  |  |  |  | miR-192-5p | up | 74 | 60 | 0.72 | | 0.817 | 0.604 | _ | _ |
|  |  |  |  |  |  |  |  |  |  |  |  |  | miR-30b-5p | down | 76 | 68 | 0.798 | | 0.883 | 0.691 | _ | _ |
|  |  |  |  |  |  |  |  |  |  |  |  |  | miR-320b | down | 84 | 78 | 0.922 | | 0.97 | 0.833 | _ | _ |
|  |  |  |  |  |  | Chronic pancratitis, who underwent surgery for pain relief | 55.65±11.67 | 34.02±10.35 | serum | serum | 50 | 50 | miR-215-5p | up | 72 | 72 | 0.763 | | 0.839 | 0.664 | _ | _ |
|  |  |  |  |  |  |  |  |  |  |  |  |  | miR-122-5p | up | 98 | 56 | 0.674 | | 0.76 | 0.568 | _ | _ |
|  |  |  |  |  |  |  |  |  |  |  |  |  | miR-192-5p | up | 70 | 54 | 0.721 | | 0.805 | 0.621 | _ | _ |
|  |  |  |  |  |  |  |  |  |  |  |  |  | miR-30b-5p | down | 70 | 56 | 0.666 | | 0.76 | 0.568 | _ | _ |
|  |  |  |  |  |  |  |  |  |  |  |  |  | miR-320b | down | 100 | 100 | 1 | | 0.054 | 0.002 | _ | _ |
| 18 | Wang, 2021 | China | Case-control | PC | Pca patients undergoing neoadjuvant therapy were excluded. | HC | _ | _ | serum | serum | 62 | 53 | miR-19b-3p | down | 85.48 | 90.57 | 0.942 | 0.977 | 0.883 | 0.001 | _ | _ |
|  |  |  |  |  |  | Chronic pancreatitis |  |  |  |  | 62 | 23 | miR-19b-3p | down | 80.65 | 86.96 | 0.898 | 0.953 | 0.813 | 0.001 | _ | _ |
|  |  |  |  |  |  | Other pancreatic tumor: pancreatic neuroendocrine tumor, solid pseudopapillary tumor, serous or mucinous cystadenomas, intraductal papillary mucinous neoplasms, and epithelial cysts. |  |  |  |  | 62 | 30 | miR-19b-3p | down | 93.55 | 63.33 | 0.81 | 0.884 | 0.714 | 0.001 | _ | _ |
| 19 | Alemar, 2016 | Brazil | cohort | PDAC | pathology-proven diagnosis and no history of previous or current chemotherapeutic or radiotherapeutic treatments. | HC, no clinical evidence of pancreatic and/or biliary disease and had a normal abdominal computed tomography scan with contrast (within a 12-month period from the recruitment date) | 62 (43–85) |  | serum | serum | 24 | 9 | mir-34a | up | 91.3 | 77.8 | 0.865 | 1 | 0.719 | 0.002 | _ | _ |
|  |  |  |  |  |  |  |  |  |  |  |  |  | mir-34a | up | 91.3 | 77.8 | 0.865 | 1 | 0.719 | 0.002 | _ | _ |
|  |  |  |  |  |  |  |  |  |  |  |  |  | mir-155 | up (not significance) | _ | _ | 0.343 | 0.572 | 0.114 | 0.169 | _ | _ |
|  |  |  |  |  |  |  |  |  |  |  |  |  | mir-196a | undetectable | _ | _ | 0.286 | 0.514 | 0.055 | 0.079 | _ | _ |
|  |  |  |  |  |  |  |  |  |  |  |  |  | mir-200b | undetectable | _ | _ | 0.652 | 0.847 | 0.456 | 0.192 | _ | _ |
|  |  |  |  |  |  |  |  |  |  |  |  |  | mir-376a | same | _ | _ | 0.606 | 0.842 | 0.37 | 0.301 | _ | _ |
| 20 | Álvarez-Hilario, 2023 | Mexico | cohort | PDAC | Individuals diagnosed with PDAC without treatment, who had not suffered from pancreatitis or diabetes mellitus, and who did not have a tumor other than PDAC | HC, control subjects were those with no history of cancer, diabetes, or pancreatitis | 63 (41–83) | 46 (27–62) | serum | serum | 46 | 20 | mir-222-3p | Up | 73.91 | 80 | 0.7266 |  |  | 0.0036 |  |  |
|  |  |  |  |  |  |  |  |  |  |  |  |  | mir-345-5p | Up | 67.39 | 70 | 0.7049 | _ | _ | 0.0085 | _ | _ |
|  |  |  |  |  |  |  |  |  |  |  |  |  | mir-100-5p | Up | 39.13 | 95 | 0.7038 | _ | _ | 0.0089 | _ | _ |
|  |  |  |  |  |  |  |  |  |  |  |  |  | mir-221-3p | Up | 63.04 | 80 | 0.7364 | _ | _ | 0.0024 | _ | _ |
|  |  |  |  |  |  |  |  |  |  |  |  |  | mir-222-3p/mir-345-5p | Up | 59.78 | 85 | 0.7231 | _ | _ | <0.0001 | _ | _ |
|  |  |  |  |  |  |  |  |  |  |  |  |  | mir-222-3p/mir-100-5p | Up | 67.39 | 75 | 0.7258 | _ | _ | <0.0001 | _ | _ |
|  |  |  |  |  |  |  |  |  |  |  |  |  | mir-222-3p/mir-221-3p | Up | 72.83 | 72.5 | 0.7207 | _ | _ | <0.0001 | _ | _ |
|  |  |  |  |  |  |  |  |  |  |  |  |  | mir-345-5p/mir-100-5p | Up | 53.26 | 80 | 0.6957 | _ | _ | 0.0004 | _ | _ |
|  |  |  |  |  |  |  |  |  |  |  |  |  | mir-345-5p/mir-221-3p | Up | 43.48 | 90 | 0.7087 | _ | _ | 0.0001 | _ | _ |
|  |  |  |  |  |  |  |  |  |  |  |  |  | mir-100-5p/mir-221-3p | Up | 51.09 | 85 | 0.7174 | _ | _ | <0.0001 | _ | _ |
|  |  |  |  |  |  |  |  |  |  |  |  |  | mir-222-3p/mir-345-5p/mir-100-5p | Up | 61.11 | 80 | 0.7159 | _ | _ | <0.0001 | _ | _ |
|  |  |  |  |  |  |  |  |  |  |  |  |  | mir-222-3p/mir-345-5p/mir-221-3p | Up | 64.5 | 75 | 0.7158 | _ | _ | <0.0001 | _ | _ |
|  |  |  |  |  |  |  |  |  |  |  |  |  | mir-100-5p/mir-221-3p/mir-222-3p | Up | 71 | 66.67 | 0.721 | _ | _ | <0.0001 | _ | _ |
|  |  |  |  |  |  |  |  |  |  |  |  |  | mir-100-5p/mir-221-3p/mir-345-5p | Up | 42 | 90 | 0.7158 | _ | _ | <0.0001 | _ | _ |
|  |  |  |  |  |  |  |  |  |  |  |  |  | mir-222-3p/ mir-100-5p/mir-221-3p/mir-345-5p | Up | 66.3 | 76.25 | 0.7189 | _ | _ | <0.0001 | _ | _ |
| 21 | Xu, 2021 | China | Case-control | PC | initial diagnosis of tumors, and pathological diagnoses were subsequently confirmed. | HC | ≥ 65: 26 | _ | serum | serum | 46 | 50 | mir-1290 | Up | 60.9 | 90 | 0.8857 | _ | _ | <0.0001 | _ | _ |
| 22 | Satu, 2020 | Japan | Case-control | IPMN (11 patients with IPMC and 27 patients with benign IPMN) | diagnosed with IPMNs by imaging modalities, i) Dilation of the MD and/or a cystic dilation of the BD, and ii) secretion of mucin from the major or minor papilla identified by endoscopic retrograde cholangiopancreatography or duodenoscopy. | controls without any type of tumor | 74.0 (47.091.0) | 71.0 (46.089.0) | serum | serum | 38 | 21 | mir-22 | up(not significant) | 78.9 | 47.6 | 0.59 | 0.76 | 0.42 | _ | _ | _ |
|  |  |  |  |  |  |  |  |  |  |  |  |  | mir-4539 | Up | 60.5 | 95.2 | 0.72 | 0.85 | 0.59 | _ | _ | _ |
|  |  |  |  |  |  |  |  |  |  |  |  |  | mir-6132 | up(not significant) | 76.2 | 66.7 | 0.64 | 0.81 | 0.47 | _ | _ | _ |
|  |  |  |  | benign IPMN | _ | IPMC | 69.5 (48.0‑89.0) | 74.5 (47.0‑91.0) | serum | serum | 27 | 11 | mir-22 | up(not significant) | 58.3 | 69.2 | 0.61 | 0.81 | 0.41 | _ | _ | _ |
|  |  |  |  |  |  |  |  |  |  |  |  |  | mir-4539 | up(not significant) | 100 | 38.4 | 0.58 | 0.77 | 0.4 | _ | _ | _ |
|  |  |  |  |  |  |  |  |  |  |  |  |  | miR6132 | up | 88.3 | 55.4 | 0.77 | 0.93 | 0.61 | _ | _ | _ |
| 23 | Jiang, 2021 | China | Case-control | PDAC | have not received any preoperative antineoplastic therapy | Healthy people | 52.74 ± 8.05 | 50.95 ± 7.39 | serum | serum | 184 | 184 | miR-607 | down | 64.7 | 77.2 | 0.785 | 0.831 | 0.738 | <0.001 | _ | _ |
|  |  |  |  |  |  | adjacent normal tissues | 56.12 ± 6.24 | 56.12 ± 6.24 | Tissue | Tissue | 50 | 50 | miR-607 | down | 63.9 | 75.5 | 0.749 | 0.845 | 0.653 | <0.001 | _ | _ |
| 24 | Liu, 2012 | China | Cohort | PDAC |  | HC, The health condition checkup included a detailed history; physical, radiological, and endoscopic examinations; blood tests; tumor marker tests (CA19-9, CEA); and abdominal sonography. Subjects with no evidence of pancreatic disease or other abnormalities were enrolled as cancer-free controls. | 62.57 (13.23) | 58.39 (12.24) | serum | serum | 25 | 25 | miR-20a, miR-21, miR-24, miR-25, miR-99a, miR-185, miR-191 | Up | 0.94 | 0.93 | 0.992 (0.027) | _ | _ | _ | _ | _ |
|  |  |  |  |  |  | HC, The health condition checkup included a detailed history; physical, radiological, and endoscopic examinations; blood tests; tumor marker tests (CA19-9, CEA); and abdominal sonography. Subjects with no evidence of pancreatic disease or other abnormalities were enrolled as cancer-free controls. | 62.57 (13.23) | 58.39 (12.24) | serum | serum | 95 | 81 | miR-20a, miR-21, miR-24, miR-25, miR-99a, miR-185, miR-191 | Up | 0.89 | 1 | 0.985 (0.021) | _ | _ | _ | _ | _ |
|  |  |  |  |  |  | CP, at least 1 of the following criteria: (a) substantial changes in the pancreatogram shown by endoscopic retrograde cholangiopancreatography (ERCP); (b) pancreatic calcification shown by computed tomography or endoscopic ultrasonography (EUS); and (c) clinical presentation of abdominal pain with increases in serum pancreatic enzymes for at least 6 months. The absence of coexisting PaC was confirmed by EUS or ERCP. | 62.57 (13.23) | 44.75 (15.46) | serum | serum | 120 | 82 | miR-20a, miR-21, miR-24, miR-25, miR-99a, miR-185, miR-191 | Up | _ | _ | 0.993 (0.008) | _ | _ | _ | _ | _ |
| 25 | Makler, 2023 | USA | Case-control | PDAC | _ | HC | 70 | 44.17 | serum | serum | 15 | 19 | miR-93-5p, miR-339-3p, miR-425-5p, and miR-425-3p |  | 80 | 94.7 | 0.885 | 1 | 0.74 | p < 1 × 10−10 | _ | _ |
| 26 | Traeger, 2018 | Germany | Case-control | PDAC |  | HC, age-matched healthy controls | Stage II:65.6 (42.4– 81.9),stage III:75.0 (49.9– 82.4), and stage IV:69.5 (35.2– 80.3) | 62.7 (42.5– 87.9) | Tissue | Tissue | 65 | 34 | miR-205 | Up | _ | _ | 0.611 | 0.771 | 0.451 | 0.239 | _ | _ |
|  |  |  |  |  |  | CP |  | 60.4 (34.0– 80.3) |  |  | 65 | 32 | miR-205 | Up | _ | _ | 0.732 | 0.87 | 0.594 | 0.007 | _ | _ |
|  |  |  |  |  |  | Non-PDAC, (age-matched healthy controls, patients with chronic pancreatitis, and patients with IPMN as a pre-cancerous lesion of the pancrea) |  | _ |  |  | 65 | 77 | miR-205 | Up | 58.1 | 75.6 | 0.673 | 0.809 | 0.537 | 0.012 | _ | _ |
|  |  |  |  |  |  | HC, age-matched healthy controls |  | 62.7 (42.5– 87.9) | Serum | Serum | 65 | 43 | miR-205 | Up | _ | _ | 0.722 | 0.887 | 0.557 | 0.008 | _ | _ |
|  |  |  |  |  |  | CP |  | 60.4 (34.0– 80.3) |  |  | 65 | 32 | miR-205 | Up | _ | _ | 0.671 | 0.825 | 0.517 | 0.051 | _ | _ |
|  |  |  |  |  |  | Non-PDAC, (age-matched healthy controls, patients with chronic pancreatitis, and patients with IPMN as a pre-cancerous lesion of the pancrea) |  | _ |  |  | 65 | 77 | miR-205 | Up | 64.3 | 68.4 | 0.669 | 0.789 | 0.548 | 0.01 | _ | _ |
| 27 | Liu 2020 | China | Case-control | PDAC | Inclusion criteria : (1) first-time diagnosis; (2) no prior history of radiotherapy, chemotherapy, and other adjuvant therapy; and (3) no other malignant tumors | healthy control | 58 (10) | 60 (11) | plasma | plasma | 40 | 40 | miR-181b | up | 77.5 | 85 | 0.789 | 0.898 | 0.681 | P < 0.05 | _ | _ |
|  |  |  |  |  |  |  |  |  |  |  |  |  | miR-196a | up | 72.5 | 92.5 | 0.865 | 0.951 | 0.779 | P < 0.05 | _ | _ |
|  |  |  |  |  |  |  |  |  |  |  |  |  | miR-210 | up | 82.5 | 80 | 0.834 | 0.923 | 0.745 | P < 0:05 | _ | _ |
|  |  |  |  |  |  |  |  |  |  |  |  |  | miR-181b+miR-196a | up | 92.50 | 90.00 | 0.944 | 1.001 | 0.887 | <0.0001 | _ | _ |
|  |  |  |  |  |  |  |  |  |  |  |  |  | miR-181b+miR-210 | up | 80.00 | 70.00 | 0.83 | 0.917 | 0.743 | <0.0001 | _ | _ |
|  |  |  |  |  |  |  |  |  |  |  |  |  | miR-196a+miR-210 | up | 87.50 | 80.00 | 0.888 | 0.970 | 0.805 | <0.0001 | _ | _ |
|  |  |  |  |  |  |  |  |  |  |  |  |  | miR-181b+196a+210 | up | 95.00 | 97.50 | 0.968 | 1.011 | 0.924 | <0.0001 | _ | _ |
| 28 | Yu 2020 | China | Case-control | pancreatic adenocarcinoma | The enrollment criteria were: (1)  pathologically confirmed pancreatic adenocarcinoma; (2) no history of other primary malignancies;(3) have not received any treatment. The exclusion criteria were: (1) incomplete clinic pathological data; (2) received chemotherapy or radiotherapy; and (3) acute inflammatory disease, including diseases that can cause secondary diabetes, such as hepatogenic diabetes, Cushing’s syndrome, glucagonoma, pheochromocytoma, hyperthyroidism and somatostatin, and other types of diabetes, high blood sugar caused by drugs, etc. | healthy control of matched gender and age without any signs or history of cancer before.Health examination, including serum carcinoembryonic antigen (CEA), carbohydrate antigen 19-9 (CA19-9) and carbohydrate antigen 125 (CA125) and plasma Total Bilirubin (TBIL) test, as well as chest X-ray were conducted to make sure no signs of diseases. Among all 91 normal controls, none of them had been diagnosed with any type of cancer before. | 62.26 (9.51) | 59.98 (7.14) | serum | serum | 80 | 91 | miR-25 | up | 82.5 | 93.64 | 0.939 | 0.975 | 0.903 | P<0.01 | _ | _ |
| 29 | Morimura 2011 | Japan | Case-control | PC (adenosquamous carcinoma(n=2), anaplastic carcinoma(n=1), adenocarcinoma(n=33)) | were pathologically diagnosed as having pancreatic cancer using surgical specimens and biopsies. | medical personnel and patients with a benign disease such as cholelithiasis.They underwent medical examinations and did not have any pancreatic disease or other cancerous disease | 64.66 (31.11) | _ | plasma | plasma | 36 | 30 | miR-18a | up | _ | _ | 0.9369 | _ | _ | P<0.0001 | _ | _ |
| 30 | Sato 2020 | Japan | Case-control | Intraductal papillary mucinous neoplasm-derived carcinoma | _ | Benign Intraductal papillary mucinous neoplasm | 74.5 (32.59) | 68.83 (30.37) | serum | serum | 11 | 28 | EVmiR22 | up | 78.9 | 47.6 | 0.59 | 0.81 | 0.41 | p=0.28 | _ | _ |
|  |  |  |  |  |  |  |  |  |  |  |  |  | EVmiR4539 | up | 0.58 | 100 | 38.4 | 0.77 | 0.4 | p=0.39 | _ | _ |
|  |  |  |  |  |  |  |  |  |  |  |  |  | EVmiR6132 | up | 88.3 | 65.4 | 0.77 | 0.93 | 0.61 | p=0.007 | _ | _ |
| 31 | Wang 2009 | USA | case-control | PDAC | Pathology confirmed | healthy spouses, friends, or nonblood relatives of patients with various nongastrointestinal and nonsmoking-related cancers | – | – | plasma | plasma | 49 | 36 | miR-21 | up | 46 | 89 | 0.63 | 0.75 | 0.51 | _ | _ | _ |
|  |  |  |  |  |  |  |  |  |  |  | 44 | 34 | miR-210 | up | 42 | 73 | 0.62 | 0.74 | 0.49 | _ | _ | _ |
|  |  |  |  |  |  |  |  |  |  |  | 39 | 29 | miR-155 | up | 53 | 78 | 0.6 | 0.74 | 0.46 | _ | _ | _ |
|  |  |  |  |  |  |  |  |  |  |  | 31 | 24 | miR-196a | up | 43 | 84 | 0.66 | 0.8 | 0.51 | _ | _ | _ |
| 32 | Xu 2016 | China | Case-control | PC | Pancreatic cancer was diagnosed based on cytological or histological examinations. | healthy volunteers | _ | _ | plasma | plasma | 156 | 65 | MiR-486-5p | up | 75 | 87.7 | 0.861 | 0.904 | 0.808 | P< 0.0001 | _ | _ |
|  |  |  |  |  |  |  |  |  |  |  |  |  | miR-126- 3p | up | 61.5 | 60 | 0.618 | 0.682 | 0.55 | P= 0.0044 | _ | _ |
|  |  |  |  |  |  |  |  |  |  |  |  |  | miR-938 | up | 61.5 | 73.8 | 0.693 | 0.753 | 0.628 | P< 0.0001 | _ | _ |
|  |  |  |  |  |  |  |  |  |  |  |  |  | miR-181c-5p | up |  |  | 0.507 |  |  | 0.8675 | _ | _ |
|  |  |  |  |  |  |  |  |  |  |  |  |  | miR-26b-3p | up |  |  | 0.564 |  |  | 0.1197 | _ | _ |
|  |  |  |  |  |  | pancreatic neuroendocrine tumors (PNET) | _ | _ | plasma | plasma | 156 | 27 | miR-938 | up | 30.8 | 100 | 0.66 | 0.728 | 0.586 | 0.0023 | _ | _ |
|  |  |  |  |  |  |  |  |  |  |  |  |  | miR-126–3p | up | 57.7 | 74.1 | 0.641 | 0.71 | 0.567 | 0.0083 | _ | _ |
|  |  |  |  |  |  |  |  |  |  |  |  |  | miR-19b-3p | up | 35.9 | 92.6 | 0.638 | 0.708 | 0.564 | 0.0062 | _ | _ |
|  |  |  |  |  |  |  |  |  |  |  |  |  | miR-26b-3p | up | 68.6 | 59.3 | 0.639 | 0.709 | 0.565 | 0.0116 | _ | _ |
|  |  |  |  |  |  | other panceratic cancer (OPT ):included serous or mucinous cystadenomas, solid pseudopapillary tumors, intraductal papillary mucinous neoplasms, or epithelial cysts | _ | _ | ؟ | ? | 156 | 58 | miR-938 | up | 84 | 35.1 | 0.618 | 0.683 | 0.549 | 0.0063 | _ | _ |
|  |  |  |  |  |  |  |  |  |  |  |  |  | miR-19b-3p | up | _ | _ | 0.533 |  |  | 0.4382 | _ | _ |
|  |  |  |  |  |  |  |  |  |  |  |  |  | miR-26b-3p | up | _ | _ | 0.565 |  |  | 0.1162 | _ | _ |
|  |  |  |  |  |  |  |  |  |  |  |  |  | miR-181c-5p | up | _ | _ | 0.517 |  |  | 0.6724 | _ | _ |
|  |  |  |  |  |  | Chronic pancreatitis was diagnosed on the basis of clinical diagnostic criteria or histological examinations. | _ | _ | blood | blood | 156 | 57 | miR-663b | down | 75 | 47.4 | 0.607 | 0.673 | 0.538 | p=0.0174 | _ | _ |
|  |  |  |  |  |  |  |  |  |  |  |  |  | miR-938 | up | 75.6 | 64.9 | 0.754 | 0.811 | 0.691 | p<0.0001 | _ | _ |
|  |  |  |  |  |  |  |  |  |  |  |  |  | miR-126–3p | up | 55.8 | 68.4 | 0.612 | 0.678 | 0.543 | p=0.0083 | _ | _ |
|  |  |  |  |  |  |  |  |  |  |  |  |  | miR-486–5p | up | 74.4 | 66.7 | 0.706 | 0.766 | 0.639 | p<0.0001 | _ | _ |
|  |  |  |  |  |  |  |  |  |  |  |  |  | miR-19b-3p | up | 52.6 | 71.9 | 0.585 | 0.652 | 0.515 | p=0.0385 | _ | _ |
|  |  |  |  |  |  |  |  |  |  |  |  |  | miR-26b-3p | up | 35.3 | 84.2 | 0.57 | _ | _ | p=0.0899 | _ | _ |
| 33 | Yu 2017 | China | Case-control | pancreatic ductal adenocarcinoma | They were confirmed by either pathological examination or fine-needle aspiration cytology (during surgery or by EUS) | healthy individuals who are all confirmed with healthy conditions after undergoing blood tests and imaging examinations in the Department of Cancer Prevention | 48.82 (9.75) | 44.65 (6.85) | plasma | plasma | 31 | 28 | miR-21 | up |  |  | 0.845 | 0.949 | 0.74 |  |  |  |
|  |  |  |  |  |  |  |  |  |  |  |  |  | miR-210 | up | _ | _ | 0.687 | 0.831 | 0.543 | _ | _ | _ |
|  |  |  |  |  |  |  |  |  |  |  |  |  | miR-155 | up | _ | _ | 0.822 | 0.937 | 0.707 | _ | _ | _ |
|  |  |  |  |  |  |  |  |  |  |  |  |  | miR-196a | up | _ | _ | 0.791 | 0.916 | 0.665 | _ | _ | _ |
|  |  |  |  |  |  |  |  |  |  |  |  |  | miR-20a | up | _ | _ | 0.884 | 0.978 | 0.79 | _ | _ | _ |
|  |  |  |  |  |  |  |  |  |  |  |  |  | miR-25 | up | _ | _ | 0.763 | 0.891 | 0.635 | _ | _ | _ |
| 34 | Xie 2014 | China | case-control | PC: ductal adenocarcinoma(n=36), acinar cell carcinoma(n=3), mucinous cystadenocarcinoma(n=1) | All patient histopathology results were confirmed by pathology after surgical tumor resection, and no concurrent oral, systemic, or infectious diseases, such as hepatitis or systemic lupus erythematosus, were diagnosed. Patients with a diagnosis of other malignancies and those receiving chemotherapy and radiotherapy before saliva collection were excluded | benign pancreatic tumor confirmed by pathology | 60.3 (13) | 51.3 (13.2) | Saliva | Saliva | 40 | 20 | miR-3679-5p | down | 90 | 45 | 0.716 | _ | _ | 0.007 | _ | _ |
|  |  |  |  |  |  |  |  |  |  |  | 40 | 20 | miR-940 | up | 62.5 | 75 | 0.729 | _ | _ | 0.004 | _ | _ |
|  |  |  |  |  |  |  |  |  |  |  | 40 | 48 | miR-3679-5p | down | 82.5 | 45 | 0.973 | _ | _ | 0.008 | _ | _ |
|  |  |  |  |  |  |  |  |  |  |  | 40 | 48 | miR-940 | up | 90 | 40 | 0.68 | _ | _ | 0.006 | _ | _ |
| 35 | Wang 2014 | USA | Case-control | PDAC | on the basis of clinical history and laboratory, radiologic (computed tomography or magnetic resonance imaging), and cytologic or histologic findings | Non-pancreatic, non-healthy controls: Patients with no prior history of pancreatic disease and who had normal pancreatic test results (ultrasound, cholangiopancreatography, computed tomography, or magnetic resonance imaging) were deemed to have normal pancreata | _ | _ | pancreatic juice | pancreatic juice | 50 | 19 | miR-205 | up | 64 | 74 | 0.68 | 0.83 | 0.53 | _ | _ | _ |
|  |  |  |  |  |  |  |  |  |  |  |  |  | miR-210 | up | 76 | 95 | 0.84 | 0.94 | 0.75 | _ | _ | _ |
|  |  |  |  |  |  |  |  |  |  |  |  |  | miR-492 | up | 73 | 82 | 0.8 | 0.68 | 0.92 | _ | _ | _ |
|  |  |  |  |  |  |  |  |  |  |  |  |  | miR-1247 | up | 53 | 88 | 0.63 | 0.5 | 0.77 | _ | _ | _ |
| 36 | Vychytilova-Faltejskova 2015 | Czech Republic | Case-control | PDAC | – | chronic pancreatitis | 56.5 (36.29) | – | tissue | tissue | 74 | 18 | miR-21 | up | 93.24 | 72.22 | 0.9227 | _ | _ | p<0.0001 | _ | _ |
|  |  |  |  |  |  |  |  |  |  |  |  |  | miR-198 | up | 81.08 | 72.22 | 0.7748 | _ | _ | p=0.0003 | _ | _ |
|  |  |  |  |  |  |  |  |  |  |  |  |  | miR-34a | up | 86.67 | 61.11 | 0.82 | _ | _ | p<0.0001 | _ | _ |
|  |  |  |  |  |  |  |  |  |  |  |  |  | miR-217 | down | 64.86 | 61.11 | 0.6652 | _ | _ | p=0.0304 | _ | _ |
| 37 | Shao 2021 | China | Case-control | PDAC |  | non-pathologic healthy controls | 60 ± 9 | 50 ± 8.7 | blood | blood | 63 | 22 | miR-483-3p | up | 74.6 | 77.3 | 0.81 | 0.91 | 0.71 | p<0.0001 | _ | _ |
|  |  |  |  |  |  |  |  |  | exosome | exosome |  |  | miR-483-3p | up | _ | _ | 0.69 | 0.81 | 0.58 | p<0.001 | _ | _ |
| 38 | Traeger 2018 | Germany | Case-control | PDAC | Patients that received immunosuppression, chemo- or radiotherapy before blood sampling and/or surgery were excluded | heathy controls: benign, non-inflammatory pancreatic specimens of  patients with acinar cell cystadenoma or serous cystadenoma | – | – | tissue | tissue | 34 | 17 | miR-205 | up | – | – | 0.611 | 0.771 | 0.451 | 0.239 | – | – |
|  |  |  |  |  |  |  |  |  | blood | blood | 47 | 17 | miR-205 | up | – | – | 0.722 | 0.887 | 0.557 | 0.008 | – | – |
|  |  |  |  |  |  | Chronic pancreatiti | – | – | tissue | tissue | 34 | 16 | miR-205 | up | – | – | 0.732 | 0.87 | 0.594 | 0.007 | – | – |
|  |  |  |  |  |  |  | – | – | blood | blood | 47 | 16 | miR-205 | up | – | – | 0.671 | 0.825 | 0.517 | 0.051 | – | – |
|  |  |  |  |  |  | non _ PDAC (healthy controls17+Chronic pancreatiti16+IPMN6) | – | – | tissue | tissue | 34 | 39 | miR-205 | up | 0.581 | 0.756 | 0.673 | 0.809 | 0.537 | 0.012 | – | – |
|  |  |  |  |  |  |  | – | – | blood | blood | 47 | 38 | miR-205 | up | 0.643 | 0.684 | 0.669 | 0.789 | 0.548 | 0.01 | – | – |
| 39 | Li 2013 | USA | Case-control | PDAC | preoperative samples from patients with resectable PDACs, we did not include any patients whose pancreatic cancer had caused obstructive jaundice before their pancreatic resection | Healthy individuals, none of the controls had obstructive jaundice). | – | – | serum | serum | 81 | 39 | miR-1290 | up | 88 | 84 | 0.96 | 1 | 0.91 | p< 0.001 | – | – |
|  |  |  |  |  |  |  |  |  |  |  |  |  | miR-550 | up | 73 | 58 | 0.74 | 0.87 | 0.6 | p=0.003 | – | – |
|  |  |  |  |  |  |  |  |  |  |  |  |  | miR-628-3p | up | 75 | 84 | 0.82 | 0.94 | 0.71 | <0.001 | – | – |
|  |  |  |  |  |  |  |  |  |  |  |  |  | miR-1825 | up | 63 | 79 | 0.7 | 0.85 | 0.56 | p=0.012 | – | – |
|  |  |  |  |  |  |  |  |  |  |  |  |  | miR-24 | up | 73 | 68 | 0.79 | 0.9 | 0.68 | <0.001 | – | – |
|  |  |  |  |  |  |  |  |  |  |  |  |  | miR-134 | up | 73 | 68 | 0.8 | 0.92 | 0.69 | <0.001 | – | – |
|  |  |  |  |  |  |  |  |  |  |  |  |  | miR-146a | up | 78 | 79 | 0.82 | 0.93 | 0.71 | <0.001 | – | – |
|  |  |  |  |  |  |  |  |  |  |  |  |  | miR-200c | up | 66 | 68 | 0.64 | 0.79 | 0.5 | p=0.079 | – | – |
|  |  |  |  |  |  |  |  |  |  |  |  |  | miR-378 | up | 76 | 79 | 0.81 | 0.92 | 0.69 | <0.001 | – | – |
|  |  |  |  |  |  |  |  |  |  |  |  |  | miR-484 | up | 76 | 63 | 0.78 | 0.9 | 0.66 | <0.001 | – | – |
|  |  |  |  |  |  |  |  |  |  |  |  |  | miR-625 | up | 63 | 53 | 0.66 | 0.8 | 0.51 | p=0.054 | – | – |
|  |  |  |  |  |  |  |  |  |  |  |  |  | miR-22 | up | 71 | 79 | 0.729 | 0.86 | 0.59 | p=0.005 | – | – |
|  |  |  |  |  |  |  |  |  |  |  |  |  | miR-210 | up | 73 | 58 | 0.727 | 0.86 | 0.59 | p=0.005 | – | – |
|  |  |  |  |  |  |  |  |  |  |  |  |  | miR744 | up | 68 | 53 | 0.691 | 0.84 | 0.54 | p=0.018 | – | – |
|  |  |  |  |  |  | chronic pancreatitis, none of the controls had obstructive jaundice | – | – | serum | serum | 81 | 45 | miR-1290 | up | 83 | 69 | 0.81 | 0.91 | 0.71 | <0.001 | – | – |
|  |  |  |  |  |  |  |  |  |  |  |  |  | miR-625 | up | 71 | 63 | 0.66 | 0.78 | 0.53 | 0.018 | – | – |
|  |  |  |  |  |  |  |  |  |  |  |  |  | miR-550 | up | 51 | 69 | 0.63 | 0.76 | 0.51 | 0.049 | – | – |
|  |  |  |  |  |  |  |  |  |  |  |  |  | miR-628-3p | up | 71 | 57 | 0.69 | 0.81 | 0.57 | 0.004 | – | – |
|  |  |  |  |  |  |  |  |  |  |  |  |  | miR-1825 | up | 76 | 60 | 0.71 | 0.86 | 0.56 | 0.011 | – | – |
|  |  |  |  |  |  |  |  |  |  |  |  |  | miR-24 | up | 73 | 63 | 0.7 | 0.82 | 0.57 | 0.003 | – | – |
|  |  |  |  |  |  |  |  |  |  |  |  |  | miR-134 | up | 73 | 51 | 0.66 | 0.79 | 0.54 | 0.015 | – | – |
|  |  |  |  |  |  |  |  |  |  |  |  |  | miR-146a | up | 73 | 80 | 0.78 | 0.89 | 0.68 | <0.001 | – | – |
|  |  |  |  |  |  |  |  |  |  |  |  |  | miR-200c | up | 66 | 57 | 0.66 | 0.78 | 0.53 | 0.02 | – | – |
|  |  |  |  |  |  |  |  |  |  |  |  |  | miR-378 | up | 68 | 63 | 0.67 | 0.79 | 0.54 | 0.011 | – | – |
|  |  |  |  |  |  |  |  |  |  |  |  |  | miR-484 | up | 68 | 69 | 0.75 | 0.86 | 0.64 | <0.001 | – | – |
|  |  |  |  |  |  | Chronic pancreatitis and healthy individuals | – | – | blood | blood | 81 | 84 | miR-1290 | up | 83 | 78 | 0.86 | 0.94 | 0.79 | <0.001 | – | – |
|  |  |  |  |  |  |  |  |  |  |  |  |  | miR-625 | up | 71 | 57 | 0.66 | 0.77 | 0.56 | 0.009 | – | – |
|  |  |  |  |  |  |  |  |  |  |  |  |  | miR-550 | up | 66 | 56 | 0.67 | 0.78 | 0.56 | 0.005 | – | – |
|  |  |  |  |  |  |  |  |  |  |  |  |  | miR-628-3p | up | 73 | 67 | 0.74 | 0.84 | 0.64 | <0.001 | – | – |
|  |  |  |  |  |  |  |  |  |  |  |  |  | miR-1825 | up | 68 | 65 | 0.71 | 0.85 | 0.58 | 0.011 | – | – |
|  |  |  |  |  |  |  |  |  |  |  |  |  | miR-24 | up | 66 | 76 | 0.73 | 0.83 | 0.63 | <0.001 | – | – |
|  |  |  |  |  |  |  |  |  |  |  |  |  | miR-134 | up | 66 | 63 | 0.71 | 0.81 | 0.61 | <0.001 | – | – |
|  |  |  |  |  |  |  |  |  |  |  |  |  | miR-146a | up | 78 | 70 | 0.8 | 0.89 | 0.7 | <0.001 | – | – |
|  |  |  |  |  |  |  |  |  |  |  |  |  | miR-200c | up | 66 | 61 | 0.65 | 0.76 | 0.54 | 0.012 | – | – |
|  |  |  |  |  |  |  |  |  |  |  |  |  | miR-378 | up | 71 | 70 | 0.72 | 0.82 | 0.61 | <0.001 | – | – |
|  |  |  |  |  |  |  |  |  |  |  |  |  | miR-484 | up | 76 | 63 | 0.76 | 0.86 | 0.67 | <0.001 | – | – |
|  |  |  |  |  |  | pancreatic neuroendocrine tumors (PNET), |  |  | blood | blood | 81 | 28 | miR-1290 | up | 81 | 61 | 0.80 | 0.93 | 0.67 | <0.001 | – | – |
|  |  |  |  |  |  |  |  |  |  |  |  |  | miR-628-3p | up | 73 | 56 | 0.68 | 0.83 | 0.53 | 0.031 | – | – |
|  |  |  |  |  |  |  |  |  |  |  |  |  | miR-550 | up | 61 | 66 | 0.70 | 0.84 | 0.56 | 0.015 | – | – |
|  |  |  |  |  |  |  |  |  |  |  |  |  | miR-1825 | up | 73 | 61 | 0.72 | 0.85 | 0.58 | 0.009 | – | – |
| 40 | Zhou 2018 | China | cohort | PC | histopathological verified PC patients, surgery patients without preoperative chemo radiotherapy | healthy donors | – | – | plasma | plasma | 216 | 220 | miR-122-5p | up | – | – | 0.81 | 0.859 | 0.762 | – | – | – |
|  |  |  |  |  |  |  |  |  |  |  |  |  | miR-125b-5p | up | – | – | 0.646 | 0.707 | 0.585 | – | – | – |
|  |  |  |  |  |  |  |  |  |  |  |  |  | miR-192-5p | up | – | – | 0.693 | 0.753 | 0.634 | – | – | – |
|  |  |  |  |  |  |  |  |  |  |  |  |  | miR-193b-3p | up | – | – | 0.775 | 0.828 | 0.722 | – | – | – |
|  |  |  |  |  |  |  |  |  |  |  |  |  | miR-221-3p | up | – | – | 0.625 | 0.688 | 0.562 | – | – | – |
|  |  |  |  |  |  |  |  |  |  |  |  |  | miR-27b-3p | up | – | – | 0.656 | 0.718 | 0.594 | – | – | – |
|  |  |  |  |  |  |  |  |  |  |  |  |  | six-miRNA signature | – | 73.2 | 81.4 | 0.831 | 0.879 | 0.784 | – | – | – |
|  |  |  |  |  |  |  |  |  |  |  | 31 | 37 | miR-122-5p | up | – | – | 0.722; | 0.591 | 0.583 | – | – | – |
|  |  |  |  |  |  |  |  |  |  |  |  |  | miR-193b-3p | up | – | – | 0.651 | 0.792 | 0.51 | – | – | – |
|  |  |  |  |  |  |  |  |  |  |  |  |  | two-miRNA signature | – | – | – | 0.849 | 0.942 | 0.756 | – | – | – |
| 41 | Vila-Navarro 2016 | Spain | cohort | PDAC | None of the patients with PDAC had received chemo or radiotherapy before sample collection | Normal pancreatic tissue is from patients who underwent surgery for other reasons (ie, ampulloma or cystadenoma) or from an adjacent nontumoral part of PDAC lesions. | – | – | tissue | tissue | 60 | 26 | hsa.miR.93 | up | 100 | 96 | 0.995 | 1 | 0.99 | 3.78E-13 | 98 | 100 |
|  |  |  |  |  |  |  |  |  |  |  |  |  | hsa.miR.16 | up | 96 | 95 | 0.991 | 1 | 0.98 | 6.18E-13 | 89 | 98 |
|  |  |  |  |  |  |  |  |  |  |  |  |  | hsa.miR.548d.3p | up | 93 | 96 | 0.985 | 1 | 0.97 | 1.08E-12 | 86 | 98 |
|  |  |  |  |  |  |  |  |  |  |  |  |  | hsa.miR.320a | up | 90 | 92 | 0.961 | 0.98 | 0.94 | 1.31E-11 | 80 | 96 |
|  |  |  |  |  |  |  |  |  |  |  |  |  | hsa.miR.4468 | up | 88 | 92 | 0.954 | 0.97 | 0.93 | 2.73E-11 | 77 | 96 |
|  |  |  |  |  |  |  |  |  |  |  |  |  | hsa.miR.3120.3p | up | 92 | 88 | 0.952 | 0.97 | 0.93 | 3.28E-11 | 82 | 95 |
|  |  |  |  |  |  |  |  |  |  |  |  |  | hsa.miR.4713.5p | up | 85 | 100 | 0.947 | 0.97 | 0.93 | 5.38E-11 | 74 | 100 |
|  |  |  |  |  |  |  |  |  |  |  |  |  | hsa.miR.103a | up | 90 | 88 | 0.94 | 0.96 | 0.93 | 1.04E-10 | 79 | 95 |
|  |  |  |  |  |  |  |  |  |  |  |  |  | hsa.miR.181a | up | 92 | 88 | 0.927 | 0.95 | 0.9 | 3.82E-10 | 82 | 95 |
|  |  |  |  |  |  |  |  |  |  |  |  |  | hsa.miR.155 | up | 97 | 85 | 0.925 | 0.95 | 0.9 | 4.65E-10 | 92 | 94 |
|  |  |  |  |  |  |  |  |  |  |  |  |  | hsa.miR.4770 | up | 97 | 85 | 0.925 | 0.95 | 0.9 | 4.69E-10 | 92 | 94 |
|  |  |  |  |  |  |  |  |  |  |  |  |  | hsa.miR.221 | up | 85 | 88 | 0.912 | 0.94 | 0.88 | 1.53E-09 | 72 | 94 |
|  |  |  |  |  |  |  |  |  |  |  |  |  | hsa.miR.151b | up | 83 | 88 | 0.91 | 0.94 | 0.88 | 1.87E-09 | 69 | 94 |
|  |  |  |  |  |  |  |  |  |  |  |  |  | hsa.miR.21 | up | 83 | 88 | 0.897 | 0.93 | 0.87 | 5.52E-09 | 69 | 94 |
|  |  |  |  |  |  |  |  |  |  |  |  |  | hsa.miR.151.5p | up | 75 | 92 | 0.886 | 0.92 | 0.85 | 1.49E-08 | 61 | 96 |
|  |  |  |  |  |  |  |  |  |  |  |  |  | hsa.miR.181b | up | 90 | 77 | 0.882 | 0.92 | 0.85 | 2.06E-08 | 77 | 90 |
|  |  |  |  |  |  |  |  |  |  |  |  |  | hsa.miR.192 | up | 81 | 88 | 0.88 | 0.91 | 0.85 | 2.52E-08 | 67 | 94 |
|  |  |  |  |  |  |  |  |  |  |  |  |  | hsa.miR.23a | up | 77 | 92 | 0.871 | 0.91 | 0.83 | 5.48E-08 | 63 | 96 |
|  |  |  |  |  |  |  |  |  |  |  |  |  | hsa.miR.let.7f | up | 85 | 85 | 0.863 | 0.9 | 0.83 | 1.01E-07 | 71 | 93 |
|  |  |  |  |  |  |  |  |  |  |  |  |  | hsa.miR.1304 | up | 93 | 77 | 0.853 | 0.89 | 0.81 | 2.31E-07 | 73 | 90 |
|  |  |  |  |  |  |  |  |  |  |  |  |  | hsa.miR.1257 | up | 67 | 92 | 0.841 | 0.88 | 0.81 | 5.78E-07 | 54 | 95 |
|  |  |  |  |  |  |  |  |  |  |  |  |  | hsa.miR.4639.5p | up | 80 | 77 | 0.839 | 0.88 | 0.8 | 6.49E-07 | 62 | 89 |
|  |  |  |  |  |  |  |  |  |  |  |  |  | hsa.miR.33a. | up | 63 | 92 | 0.829 | 0.87 | 0.79 | 1.36E-06 | 52 | 95 |
|  |  |  |  |  |  |  |  |  |  |  |  |  | hsa.miR.3714 | up | 85 | 69 | 0.824 | 0.87 | 0.78 | 2.01E-06 | 66 | 86 |
|  |  |  |  |  |  |  |  |  |  |  |  |  | hsa.miR.let.7e | up | 77 | 81 | 0.813 | 0.86 | 0.77 | 4.52E-06 | 64 | 89 |
|  |  |  |  |  |  |  |  |  |  |  |  |  | hsa.miR.3133 | up | 83 | 65 | 0.809 | 0.85 | 0.76 | 5.89E-06 | 63 | 85 |
|  |  |  |  |  |  |  |  |  |  |  |  |  | hsa.miR.1181 | up | 76 | 73 | 0.79 | 0.84 | 0.74 | 2.16E-05 | 57 | 87 |
|  |  |  |  |  |  |  |  |  |  |  |  |  | hsa.miR.429 | up | 92 | 53 | 0.773 | 0.82 | 0.72 | 6.05E-05 | 46 | 94 |
|  |  |  |  |  |  |  |  |  |  |  |  |  | hsa.miR.761 | up | 64 | 77 | 0.718 | 0.77 | 0.66 | 1.40E-03 | 48 | 87 |
|  |  |  |  |  |  |  |  |  |  |  |  |  | hsa.miR.29a | up | 63 | 73 | 0.712 | 0.77 | 0.66 | 1.91E-03 | 46 | 84 |
|  |  |  |  |  |  |  |  |  |  |  |  |  | hsa.miR.4714.5p | up | 84 | 58 | 0.693 | 0.75 | 0.64 | 4.76E-03 | 62 | 82 |
|  |  |  |  |  |  |  |  |  |  |  |  |  | hsa.miR.let.7c | up | 85 | 38 | 0.601 | 0.66 | 0.54 | 1.40E-01 | 52 | 76 |
|  |  |  |  | IPMN | None of the patients with PDAC had received chemo or radiotherapy before sample collection | Normal pancreatic tissue is from patients who underwent surgery for other reasons (ie, ampulloma or cystadenoma) or from an adjacent nontumoral part of PDAC lesions. | – | – | tissue | tissue | 9 | 26 | hsa.miR.93 | up | 0.89 | 0.88 | 0.905 | 0.97 | 0.84 | 3.46E-04 | 0.96 | 0.72 |
|  |  |  |  |  |  |  |  |  |  |  |  |  | hsa.miR.16 | up | 0.89 | 0.80 | 0.881 | 0.96 | 0.81 | 7.77E-04 | 0.95 | 0.61 |
|  |  |  |  |  |  |  |  |  |  |  |  |  | hsa.miR.548d.3p | up | 0.56 | 0.96 | 0.843 | 0.93 | 0.76 | 2.47E-03 | 0.86 | 0.83 |
|  |  |  |  |  |  |  |  |  |  |  |  |  | hsa.miR.320a | up | 0.67 | 0.96 | 0.867 | 0.95 | 0.79 | 1.18E-03 | 0.89 | 0.85 |
|  |  |  |  |  |  |  |  |  |  |  |  |  | hsa.miR.4468 | up | 0.78 | 0.60 | 0.682 | 0.79 | 0.58 | 1.07E-01 | 0.89 | 0.40 |
|  |  |  |  |  |  |  |  |  |  |  |  |  | hsa.miR.3120.3p | up | 0.67 | 0.88 | 0.703 | 0.81 | 0.60 | 7.31E-02 | 0.88 | 0.66 |
|  |  |  |  |  |  |  |  |  |  |  |  |  | hsa.miR.4713.5p | up | 0.56 | 0.84 | 0.682 | 0.79 | 0.58 | 1.08E-01 | 0.85 | 0.55 |
|  |  |  |  |  |  |  |  |  |  |  |  |  | hsa.miR.103a | up | 0.78 | 0.96 | 0.914 | 0.98 | 0.85 | 2.53E-04 | 0.93 | 0.87 |
|  |  |  |  |  |  |  |  |  |  |  |  |  | hsa.miR.181a | up | 0.78 | 0.96 | 0.919 | 0.98 | 0.86 | 2.18E-04 | 0.93 | 0.87 |
|  |  |  |  |  |  |  |  |  |  |  |  |  | hsa.miR.155 | up | 1 | 0.69 | 0.912 | 0.98 | 0.85 | 2.70E-04 | 1 | 0.53 |
|  |  |  |  |  |  |  |  |  |  |  |  |  | hsa.miR.4770 | up | 0.78 | 0.88 | 0.802 | 0.89 | 0.71 | 7.72E-03 | 0.92 | 0.69 |
|  |  |  |  |  |  |  |  |  |  |  |  |  | hsa.miR.221 | up | 0.67 | 0.96 | 0.846 | 0.93 | 0.76 | 2.26E-03 | 0.89 | 0.85 |
|  |  |  |  |  |  |  |  |  |  |  |  |  | hsa.miR.151b | up | 0.89 | 0.76 | 0.873 | 0.95 | 0.80 | 9.81E-04 | 0.95 | 0.56 |
|  |  |  |  |  |  |  |  |  |  |  |  |  | hsa.miR.21 | up | 0.67 | 0.96 | 0.875 | 0.95 | 0.80 | 9.21E-04 | 0.89 | 0.85 |
|  |  |  |  |  |  |  |  |  |  |  |  |  | hsa.miR.151.5p | up | 0.78 | 0.88 | 0.860 | 0.94 | 0.78 | 1.48E-03 | 0.92 | 0.69 |
|  |  |  |  |  |  |  |  |  |  |  |  |  | hsa.miR.181b | up | 0.89 | 0.80 | 0.907 | 0.97 | 0.84 | 3.25E-04 | 0.95 | 0.61 |
|  |  |  |  |  |  |  |  |  |  |  |  |  | hsa.miR.192 | up | 1 | 0.46 | 0.772 | 0.87 | 0.68 | 1.63E-02 | 1 | 0.39 |
|  |  |  |  |  |  |  |  |  |  |  |  |  | hsa.miR.23a | up | 0.89 | 0.88 | 0.889 | 0.96 | 0.82 | 5.92E-04 | 0.96 | 0.72 |
|  |  |  |  |  |  |  |  |  |  |  |  |  | hsa.miR.let.7f | up | 0.89 | 0.84 | 0.868 | 0.95 | 0.79 | 1.14E-03 | 0.96 | 0.66 |
|  |  |  |  |  |  |  |  |  |  |  |  |  | hsa.miR.1304 | up | 0.67 | 0.72 | 0.734 | 0.84 | 0.63 | 3.84E-02 | 0.86 | 0.45 |
|  |  |  |  |  |  |  |  |  |  |  |  |  | hsa.miR.1257 | up | 0.44 | 0.96 | 0.725 | 0.83 | 0.62 | 4.72E-02 | 0.83 | 0.79 |
|  |  |  |  |  |  |  |  |  |  |  |  |  | hsa.miR.4639.5p | up | 0.78 | 0.80 | 0.829 | 0.92 | 0.74 | 3.72E-03 | 0.91 | 0.57 |
|  |  |  |  |  |  |  |  |  |  |  |  |  | hsa.miR.33a. | up | 0.78 | 0.80 | 0.835 | 0.92 | 0.75 | 3.12E-03 | 0.91 | 0.57 |
|  |  |  |  |  |  |  |  |  |  |  |  |  | hsa.miR.3714 | up | 0.88 | 0.42 | 0.644 | 0.75 | 0.54 | 2.04E-01 | 0.91 | 0.34 |
|  |  |  |  |  |  |  |  |  |  |  |  |  | hsa.miR.let.7e | up | 0.89 | 0.84 | 0.850 | 0.93 | 0.77 | 2.00E-03 | 0.96 | 0.66 |
|  |  |  |  |  |  |  |  |  |  |  |  |  | hsa.miR.3133 | up | 0.56 | 0.80 | 0.700 | 0.80 | 0.60 | 7.78E-02 | 0.84 | 0.49 |
|  |  |  |  |  |  |  |  |  |  |  |  |  | hsa.miR.1181 | up | 0.88 | 0.46 | 0.647 | 0.76 | 0.54 | 1.93E-01 | 0.91 | 0.36 |
|  |  |  |  |  |  |  |  |  |  |  |  |  | hsa.miR.429 | up | 0.67 | 0.84 | 0.805 | 0.90 | 0.71 | 6.99E-03 | 0.88 | 0.59 |
|  |  |  |  |  |  |  |  |  |  |  |  |  | hsa.miR.761 | up | 0.67 | 0.80 | 0.738 | 0.84 | 0.64 | 3.55E-02 | 0.87 | 0.54 |
|  |  |  |  |  |  |  |  |  |  |  |  |  | hsa.miR.29a | up | 0.67 | 0.92 | 0.799 | 0.89 | 0.71 | 8.37E-03 | 0.89 | 0.74 |
|  |  |  |  |  |  |  |  |  |  |  |  |  | hsa.miR.4714.5p | up | 1 | 0.50 | 0.749 | 0.85 | 0.64 | 3.58E-02 | 1 | 0.38 |
|  |  |  |  |  |  |  |  |  |  |  |  |  | hsa.miR.let.7c | up | 0.89 | 0.80 | 0.842 | 0.93 | 0.76 | 2.52E-03 | 0.95 | 0.61 |
| 42 | Nakamura,2022 | Korea | cohort | PDAC | – | nondisease controls | – | – | plasma | plasma | 62 | 33 | cf-miR30c-5p | up | 65 | 88 | 0.79 | 0.85 | 0.72 | – | 60 | 91 |
|  |  |  |  |  |  |  |  |  |  |  |  |  | cf-let7e-5p | up | 64 | 69 | 0.67 | 0.75 | 0.59 | – | 51 | 79 |
|  |  |  |  |  |  |  |  |  |  |  |  |  | cf-miR340-5p | up | 57 | 84 | 0.67 | 0.75 | 0.6 | – | 51 | 87 |
|  |  |  |  |  |  |  |  |  |  |  |  |  | cf-miR223-3p | up | 77 | 58 | 0.73 | 0.81 | 0.66 | – | 57 | 77 |
|  |  |  |  |  |  |  |  |  |  |  |  |  | cf-miR26a-5p | up | 37 | 94 | 0.6 | 0.68 | 0.52 | – | 45 | 92 |
|  |  |  |  |  |  |  |  |  |  |  |  |  | cf-miR340-3p | up | 69 | 69 | 0.71 | 0.79 | 0.64 | – | 54 | 80 |
|  |  |  |  |  |  |  |  |  |  |  |  |  | cf-miR335-5p | up | 66 | 87 | 0.77 | 0.84 | 0.71 | – | 58 | 90 |
|  |  |  |  |  |  |  |  |  |  |  |  |  | cf-miR23b-3p | up | 28 | 96 | 0.57 | 0.65 | 0.49 | – | 42 | 92 |
|  |  |  |  |  |  |  |  |  |  |  |  |  | cf-miR142-3p | up | 46 | 93 | 0.65 | 0.73 | 0.58 | – | 92 | 48 |
|  |  |  |  |  |  |  |  |  |  |  |  |  | exo-miR200c-3p | up | 45 | 91 | 0.69 | 0.77 | 0.62 | – | 91 | 47 |
|  |  |  |  |  |  |  |  |  |  |  |  |  | exo-miR148a-3p | up | 84 | 37 | 0.59 | 0.67 | 0.5 | – | 71 | 56 |
|  |  |  |  |  |  |  |  |  |  |  |  |  | exo-miR216a-5p | up | 60 | 75 | 0.71 | 0.78 | 0.64 | – | 50 | 82 |
|  |  |  |  |  |  |  |  |  |  |  |  |  | exo-miR145-5p | up | 65 | 57 | 0.59 | 0.67 | 0.51 | – | 47 | 74 |
|  |  |  |  |  |  |  |  |  |  |  |  |  | exo-miR200b-3p | up | 73 | 91 | 0.87 | 0.92 | 0.81 | – | 65 | 94 |
|  |  |  |  |  |  |  |  |  |  |  |  |  | exo-miR143-3p | up | 48 | 76 | 0.63 | 0.71 | 0.55 | – | 44 | 79 |
|  |  |  |  |  |  |  |  |  |  |  |  |  | exo-miR34a-5p | up | 65 | 69 | 0.66 | 0.74 | 0.58 | – | 51 | 79 |
|  |  |  |  |  |  |  |  |  |  |  |  |  | exo-miR429 | up | 68 | 75 | 0.76 | 0.83 | 0.68 | – | 56 | 83 |
|  |  |  |  |  |  |  |  |  |  |  |  |  | exo-miR141-3p | up | 62 | 91 | 0.78 | 0.85 | 0.72 | – | 93 | 57 |
|  |  |  |  |  |  |  |  |  |  |  |  |  | exo-miR1260b | up | 60 | 60 | 0.6 | 0.69 | 0.52 | – | 74 | 45 |
|  |  |  |  |  |  |  |  |  |  |  |  |  | exo-miR145-3p | up | 68 | 88 | 0.8 | 0.86 | 0.74 | – | 91 | 60 |
|  |  |  |  |  |  |  |  |  |  |  |  |  | exo-miR216b-5p | up | 91 | 59 | 0.79 | 0.85 | 0.72 | – | 66 | 88 |
|  |  |  |  |  |  |  |  |  |  |  |  |  | exo-miR200a-3p | up | 56 | 87 | 0.76 | 0.82 | 0.69 | – | 52 | 89 |
|  |  |  |  |  |  |  |  |  |  |  |  |  | exo-miR1260a | up | 68 | 66 | 0.69 | 0.76 | 0.61 | – | 53 | 79 |
|  |  |  |  |  |  |  |  |  |  |  |  |  | exo-miR217-5p | up | 63 | 90 | 0.79 | 0.85 | 0.73 | – | 57 | 92 |
| 43 | Yang,2022 | China | case-control | PC | – | Other Pancratic Tumor | – | – | plasma | plasma | 68 | 27 | miR-7975 | up | – | – | 0.632 | 0.739 | 0.525 | 0.03 | – | – |
|  |  |  |  |  |  |  |  |  |  |  |  |  | miR-1228 | up | – | – | 0.89 | 0.966 | 0.814 | <0.0001 | – | – |
|  |  |  |  |  |  | CP | – | – |  |  |  |  | miR-130a-3p | up | – | – | 0.703 | 0.818 | 0.589 | 0.002 | – | – |
|  |  |  |  |  |  |  |  |  |  |  |  |  | miR-21-5p | up | – | – | 0.648 | 0.765 | 0.531 | 0.025 | – | – |
|  |  |  |  |  |  |  |  |  |  |  |  |  | miR-233-3p | up | – | – | 0.643 | 0.758 | 0.529 | 0.03 | – | – |
|  |  |  |  |  |  |  |  |  |  |  |  |  | miR-1228 | up | – | – | 0.869 | 0.955 | 0.783 | < 0.0001 | – | – |
| 44 | Mądro, 2023 | Poland | case-control | PDAC | – | healthy participants | 64 ± 13.3 | 45 ± 11.8 | plasma | plasma | 20 | 31 | miRNA-93 | up | 60 | 77 | 0.69 | 0.85 | 0.53 | 0.002 | – | – |
|  |  |  |  |  |  |  |  |  |  |  |  |  | miRNA-519 | up | 59 | 79 | 0.69 | 0.85 | 0.53 | 0.002 | – | – |

Supplementary Table 3. The characteristics of included studies in case of prognosis

| **ID** | **Author, year** | **Country** | **Specimen** | **PC type** | **PC def** | **Control type** | **Age case** | **Age control** | **Case N.** | **Control N.** | **miRNA** | **Up/downregulation** | **overall survival** | **Upper CI** | **Lower**  **CI** | **p**  **value** | **Progression**  **free**  **survival** | **Upper**  **CI** | **Lower**  **CI** | **p value** | **recurrent-free survival** | **Upper CI** | **Lower CI** | **p value** | **event-free survival** | **Upper CI** | **Lower CI** | **p value** | **disease-free survival** | **Upper CI** | **Lower CI** | **p value** |  |
| --- | --- | --- | --- | --- | --- | --- | --- | --- | --- | --- | --- | --- | --- | --- | --- | --- | --- | --- | --- | --- | --- | --- | --- | --- | --- | --- | --- | --- | --- | --- | --- | --- | --- |
| 1 | Zou, 2019 | China | Serum | PC | _ | healthy donors who took routine health checkup | ≤60 : 11 (36.7%) | ≤60: 13 (43.3%) | 30 | 30 | miR‐19a‐3p | Up | HR: 3.125 | 9.091 | 1.078 | 0.036 | _ | _ | _ | _ | _ | _ | _ | _ | _ | _ | _ | _ | _ | _ | _ | _ |  |
|  |  |  |  |  | _ |  |  |  |  |  | miR‐19b‐3p | Up | HR:0.657 | 1.597 | 0.27 | 0.354 | _ | _ | _ | _ | _ | _ | _ | _ | _ | _ | _ | _ | _ | _ | _ | _ |  |
|  |  |  |  |  | _ |  |  |  |  |  | miR‐223‐3p | Up | HR:1.523 | 7.311 | 0.317 | 0.599 | _ | _ | _ | _ | _ | _ | _ | _ | _ | _ | _ | _ | _ | _ | _ | _ |  |
|  |  |  |  |  | _ |  |  |  |  |  | miR‐25‐3p | Up | HR:1.811 | 6.129 | 0.535 | 0.34 | _ | _ | _ | _ | _ | _ | _ | _ | _ | _ | _ | _ | _ | _ | _ | _ |  |
| 2 | Chen, 2019 | China | Tissue | PDAC | _ | PDAC cell line | _ | _ | 50 |  | MiR-132 | Up | HR:0.309 | 0.97 | 0.098 | 0.044 | _ | _ | _ | _ | _ | _ | _ | _ | _ | _ | _ | _ | _ | _ | _ | _ |  |
| 3 | Bai, 2015 | China | Tissue | PDAC | _ | normal human pancreatic duct epithelial cell line | _ | _ | 80 |  | miR-153 | down | HR:4.123 | 5.033 | 2.456 | 0.038 | _ | _ | _ | _ | 0.039 | _ | _ | _ | _ | _ | _ | _ | _ | _ | _ | _ |  |
| 4 | Sebastian, 2021 | USA | Tissue | PDAC  (training cohort) | _ | normal Pancreas tissue | _ | _ | 85 | 85 | miR-181b/d and miR-575 | up | HR:2.29 | 4.89 | 1.08 | 0.031 | _ | _ | _ | _ | _ | _ | _ | _ | _ | _ | _ | _ | _ | _ | _ | _ |  |
|  |  |  |  | PDAC  ( validation cohort) |  |  | _ | _ | 91 | 91 | miR-181b/d and miR-575 | up | HR:1.59 | 3.04 | 0.84 | 0.016 | _ | _ | _ | _ | _ | _ | _ | _ | _ | _ | _ | _ | _ | _ | _ | _ |  |
| 5 | Caponi, 2012 | Netherlands | Tissue | pancreatic Intraductal papillary mucinous neoplasm | _ | normal pancreatic ductal tissues | _ | _ | 65 | 5 | miR-21 | _ | Low vs. high(median): 53.3 vs. 17.2 | 76.2 vs.21.8 | 30.3 vs. 12.5 | <0.01 | _ | _ | _ | _ | _ | _ | _ | _ | _ | _ | _ | _ | median: 29.9 vs. 10.9 | 32.1 vs. 15.7 | 27.6 vs. 6.1 | 0.01 |  |
|  |  |  |  |  |  |  |  |  |  |  | miR-155 | _ | Median:  41.8 vs. 25.3 | 59.8 vs. 45.9 | 23.9 vs. 4.7 | 0.96 | _ | _ | _ | _ | _ | _ | _ | _ | _ | _ | _ | _ | 27.2 vs. 15.6 | 42.2 vs. 22.9 | 12.3 vs. 8.2 | 0.69 |  |
|  |  |  |  |  |  |  |  |  |  |  | miR-101 | _ | Median:  34.3 vs.  44.8 | 48.2 vs. 92.7 | 20.5 vs. 0 | 0.87 | _ | _ | _ | _ | _ | _ | _ | _ | _ | _ | _ | _ | 16.7 vs. 17.9 | 23.5 vs. 34.7 | 9.9 vs. 1.12 | 0.73 |  |
| 6 | Chen, 2014 | China | Serum | PC | _ | Chronic Pncretitis | _ | _ | 109 | 38 | miR-182 | _ | Low vs. high HR: 2.96 | 5.92 | 1.56 | <0.001 | _ | _ | _ | _ | _ | _ | _ | _ | _ | _ | _ | _ | Low versus High: HR: 2.55 | 4.83 | 1.28 | 0.001 |  |
| 7 | Kawamura | Japan | Serum | PDAC | _ | Healthy donors | _ | _ | 55 | 20 | miR-4525 | up | HR: 4.83 | 9.39 | 1.27 | 0.018 | _ | _ | _ | _ | _ | _ | _ | _ | _ | _ | _ | _ | HR: 4.83 | 9.39 | 1.27 | 0.018 |  |
|  |  |  |  |  |  |  |  |  |  |  | miR-451a | up | HR: 3.60 | 11.31 | 1.13 | 0.023 | _ | _ | _ | _ | _ | _ | _ | _ | _ | _ | _ | _ | HR: 2.91 | 7.7 | 1.25 | 0.012 |  |
|  |  |  |  |  |  |  |  |  |  |  | miR-21 | up | HR: 3.10 | 9.1 | 1.19 | 0.031 | _ | _ | _ | _ | _ | _ | _ | _ | _ | _ | _ | _ | HR: 2.80 | 7.26 | 1.22 | 0.015 |  |
| 8 | Giovannetti,2012 | Italy | Tissue | PDAC | _ |  | _ | _ | 26 |  | miR-211 | down | HR: 2 | 4.1 | 1.1 | 0.04 | _ | _ | _ | _ | _ | _ | _ | _ | _ | _ | _ | _ | HR:  2.08 | 7.04 | 0.61 | 0.02 |  |
| 9 | Goto, 2018 | Japan | Serum | PC | _ | HC | 64.0 ± 10.1 | 57.5 ± 15.3 | 32 | 22 | mir-21 | up | median low vs. high: 846 vs 344 days |  |  | 0.0137 | _ | _ | _ | _ | _ | _ | _ | _ | _ | _ | _ | _ | _ | _ | _ | _ |  |
| 10 | Hua, 2017 | China | Serum | PC | _ | HC | _ | _ | 103 | 50 | miR-373 | down | HR: 5.21 | 8.42 | 2.12 | 0.014 | _ | _ | _ | _ | _ | _ | _ | _ | _ | _ | _ | _ | _ | _ | _ | _ |  |
| 11 | Liu, 2016 | China | Tissue | PDAC | _ | adjacent normal pancreatic tissues | _ | _ | 57 | 25 | miR-23b-3p | down |  |  |  |  | _ | _ | _ | _ | _ | _ | _ | _ | _ | _ | _ | _ | HR: 0.831 | 0.983 | 0.702 | 0.031 |  |
| 12 | Ma, 2017 | China | Serum | PC | _ | HC | _ | _ | 96 | 30 | miR-203 | Up | RR:3.26 | 5.13 | 1.34 | 0.019 | _ | _ | _ | _ | _ | _ | _ | _ | _ | _ | _ | _ | RR: 3.79 | 5.98 | 1.58 | 0.015 |  |
| 13 | Jamieson, 2012 | UK | Tissue | PDAC | _ | nontumor pancreas | _ | _ | 48 | 10 | miR-21 | Up | HR: 3.22 | 8.58 | 1.21 | 0.019 | _ | _ | _ | _ | _ | _ | _ | _ | _ | _ | _ | _ | _ | _ | _ | _ |  |
|  |  |  |  |  |  |  |  |  |  |  | miR-29c | Up | HR: 0.53 | 1.47 | 0.19 | 0.227 | _ | _ | _ | _ | _ | _ | _ | _ | _ | _ | _ | _ | _ | _ | _ | _ |  |
|  |  |  |  |  |  |  |  |  |  |  | miR-30d | Up | HR: 0.30 | 0.79 | 0.12 | 0.014 | _ | _ | _ | _ | _ | _ | _ | _ | _ | _ | _ | _ | _ | _ | _ | _ |  |
|  |  |  |  |  |  |  |  |  |  |  | miR-34a | Up | HR: 0.15 | 0.37 | 0.06 | 0.001 | _ | _ | _ | _ | _ | _ | _ | _ | _ | _ | _ | _ | _ | _ | _ | _ |  |
|  |  |  |  |  |  |  |  |  |  |  | miR-221 | Up | HR: 0.92 | 2.54 | 0.34 | 0.881 | _ | _ | _ | _ | _ | _ | _ | _ | _ | _ | _ | _ | _ | _ | _ | _ |  |
|  |  |  |  |  |  |  |  |  |  |  | miR-224 | Up | HR: 0.67 | 1.76 | 0.25 | 0.673 | _ | _ | _ | _ | _ | _ | _ | _ | _ | _ | _ | _ | _ | _ | _ | _ |  |
| 14 | Jiraskova, 2019 | Czech Republic | Tissue | PDAC | _ |  | _ | _ | 69 |  | miR-21 | Up | HR:  0.475 | 1.12 | 0.201 | 0.089 | _ | _ | _ | _ | _ | _ | _ | _ | _ | _ | _ | _ | _ | _ | _ | _ |  |
| 15 | Kong, 2010 | China | Serum | PDAC | _ | HC | _ | _ | 35 | 15 | miR-196a | up | median high vs. low:  6.1m vs.12 | 7.72 vs. 18.08 | 4.49 vs. 5.92 | 0.007 | _ | _ | _ | _ | _ | _ | _ | _ | _ | _ | _ | _ | _ | _ | _ | _ |  |
| 16 | Lee, 2013 | China | Tissue | PDAC | _ | adjacent normal tissue | _ | _ | 36 | 60 | miR-222 | Up | HR: 5.16 | 22.91 | 1.16 | 0.03 | _ | _ | _ | _ | _ | _ | _ | _ | _ | _ | _ | _ | _ | _ | _ | _ |  |
| 17 | Kubo, 2019 |  | Tissue | PDAC | _ |  | _ | _ | 59 |  | MiR-194-5p | up | HR: 3.61 | 12.87 | 1.01 | 0.047 | _ | _ | _ | _ | _ | _ | _ | _ | _ | _ | _ | _ | _ | _ | _ | _ |  |
| 18 | Álvarez-Hilario, 2023 | Mexico | serum | PDAC | Individuals diagnosed with PDAC without treatment, who had not suffered from pancreatitis or diabetes mellitus, and who did not have a tumor other than PDAC | HC, control subjects were those with no history of cancer, diabetes, or pancreatitis | 63 (41–83) | 46 (27–62) | 46 | 20 | mir-222-3p | up | HR: 1.56 | 2.035 | 1.04 | 0.03 | _ | _ | _ | _ | _ | _ | _ | _ | _ | _ | _ | _ | _ | _ | _ | _ |  |
|  |  |  |  |  |  |  |  |  |  |  | mir-345-5p | up | HR: 0.63 | 1.02 | 0.39 | 0.06 | _ | _ | _ | _ | _ | _ | _ | _ | _ | _ | _ | _ | _ | _ | _ | _ |  |
|  |  |  |  |  |  |  |  |  |  |  | mir-100-5p | up | HR: 1.34 | 2.03 | 0.89 | 0.16 | _ | _ | _ | _ | _ | _ | _ | _ | _ | _ | _ | _ | _ | _ | _ | _ |  |
|  |  |  |  |  |  |  |  |  |  |  | mir-221-3p | up | HR: 1.62 | 2.51 | 1.05 | 0.0028 | _ | _ | _ | _ | _ | _ | _ | _ | _ | _ | _ | _ | _ | _ | _ | _ |  |
|  |  |  |  |  |  |  |  |  |  |  | mir-222-3p/mir-221-3p | up | HR: 1.59 | 2.39 | 1.06 | 0.025 | _ | _ | _ | _ | _ | _ | _ | _ | _ | _ | _ | _ | _ | _ | _ | _ |  |
| 19 | Grolmusz, 2018 | Hungary | Tissue | Pancreatic neuroendocrine neoplasms (PanNENs) | _ | _ | 54.4 ± 17.3 |  | 49 | _ | miR-21 | Up | HR:1.03 | 1.05 | 1 | 0.0313 | HR: 1.03 | 1.05 | 1.01 | 0.012 | _ | _ | _ | _ | _ | _ | _ | _ | _ | _ | _ | _ |  |
|  |  |  |  |  |  |  |  |  |  |  | miR-10a | Up | HR:1.03 | 1.34 | 0.8 | 0.7997 | HR: 1.11 | 1.38 | 0.89 | 0.3556 | _ | _ | _ | _ | _ | _ | _ | _ | _ | _ | _ | _ |  |
|  |  |  |  |  |  |  |  |  |  |  | miR-106b | Up | HR:1.03 | 1.16 | 0.91 | 0.6428 | HR: 0.97 | 1.07 | 0.87 | 0.5249 | _ | _ | _ | _ | _ | _ | _ | _ | _ | _ | _ | _ |  |
| 20 | Jiang, 2021 | China | Serum | PDAC | have not received any preoperative antineoplastic therapy | Healthy people | 50.95 ± 7.39 | 50.95 ± 7.39 | 184 | 184 | miR-607 | down | RR high vs. low: 0.449 | 0.861 | 0.253 | 0.036 | _ | _ | _ | _ | _ | _ | _ | _ | _ | _ | _ | _ | _ | _ | _ | _ |  |
| 21 | Traeger, 2018 | Germany | Tissue | PDAC | _ | _ | >60: 43 | _ | 65 | _ | miR-205 | Up | median high vs. low: 20.37 m vs.  9.46 | _ | _ | 0.219 | 12.55 vs. 15.51 | _ | 0.806 | 0.219 | _ | _ | _ | _ | _ | _ | _ | _ | _ | _ | _ | _ |  |
|  |  |  |  |  |  |  |  |  |  |  | miR-205 | Up | median high vs. low: 11.93  m vs.  6.93 | _ | _ | 0.176 | 15.51 vs. 15.31 | _ | 0.836 | 0.176 | _ | _ | _ | _ | _ | _ | _ | _ | _ | _ | _ | _ |  |
| 22 | Shao, 2021 | China | Serum | PDAC | _ | _ | _ | _ | 63 | _ | miR-483-3p | Up | HR: 3.307 | 9.903 | 1.104 | 0.033 | _ | _ | _ | _ | _ | _ | _ | _ | _ | _ | _ | _ | _ | _ | _ | _ |  |
| 23 | Vychytilova-Faltejskova, 2015 | Czech Republic | Tissue | PDAC |  |  | 60.5 (30-79) |  | 74 | _ | miR-21 | Up | median: 19.1 vs. 15.2 m | _ | _ | 0.0427 | _ | _ | _ | _ | _ | _ | _ | _ | _ | _ | _ | _ | mesian: 14.2 vs. 7.5 | _ | _ | 0.0011 |  |
|  |  |  |  |  |  |  |  |  |  |  | miR-198 | Up | median: 22.6 vs. 15.2 m | _ | _ | 0.0097 | _ | _ | _ | _ | _ | _ | _ | _ | _ | _ | _ | _ | mesian: 15.3 vs. 9.5 | _ | _ | 0.0001 |  |
|  |  |  |  |  |  |  |  |  |  |  | miR-34a | Up | median: 14.9 vs. 16.9 m | _ | _ | 0.932 | _ | _ | _ | _ | _ | _ | _ | _ | _ | _ | _ | _ | mesian: 9.6 vs. 11.5 | _ | _ | 0.7186 |  |
|  |  |  |  |  |  |  |  |  |  |  | miR-217 | down | median: 14.9 vs. 21.5 m | _ | _ | 0.137 | _ | _ | _ | _ | _ | _ | _ | _ | _ | _ | _ | _ | median: 9.6 vs. 12.2 m | _ | _ | 0.1716 |  |
|  |  |  |  |  |  |  |  |  |  |  | miR-21/miR-198 | _ | median: 23.7 vs. 14.9 m | _ | _ | 0.0015 | _ | _ | _ | _ | _ | _ | _ | _ | _ | _ | _ | _ | median: 18.2 vs. 8 m | _ | _ | <0.0001 |  |
| 24 | wang, 2014 | china | Pancreatic Juice | PDAC | _ | _ | _ | _ | 50 | _ | miR-205 | Up | HR: 1.46 | 3.12 | 0.66 | 0.347 | _ | _ | _ | _ | _ | _ | _ | _ | _ | _ | _ | _ | _ | _ | _ | _ |  |
|  |  |  |  |  |  |  |  |  |  |  | miR-210 | Up | HR: 1.78 | 4.8 | 0.66 | 0.254 | _ | _ | _ | _ | _ | _ | _ | _ | _ | _ | _ | _ | _ | _ | _ | _ |  |
|  |  |  |  |  |  |  |  |  |  |  | miR-492 | Up | HR: 0.82 | 1.83 | 0.36 | 0.62 | _ | _ | _ | _ | _ | _ | _ | _ | _ | _ | _ | _ | _ | _ | _ | _ |  |
|  |  |  |  |  |  |  |  |  |  |  | miR-1247 | Up | HR: 1.47 | 3.23 | 0.67 | 0.334 | _ | _ | _ | _ | _ | _ | _ | _ | _ | _ | _ | _ | _ | _ | _ | _ |  |
|  |  |  |  |  |  |  |  |  |  |  | miR-205/miR -210/ miR-492/miR -1247 | _ | HR: 2.87 | 8.87 | 0.93 | 0.067 | _ | _ | _ | _ | _ | _ | _ | _ | _ | _ | _ | _ | _ | _ | _ | _ |  |
|  |  |  |  |  |  |  |  |  |  |  | miR-205/miR -210 | _ | HR: 2.14 | 4.29 | 1.06 | 0.03 | _ | _ | _ | _ | _ | _ | _ | _ | _ | _ | _ | _ | _ | _ | _ | _ |  |
| 25 | Zhou, 2018 | china | Tissue | PC | _ | _ | >60 VS. ≤60: HR: 1.104 (0.779,1.565) | _ | 216 | _ | miR-122-5p | down | HR:0.728 | 1.036 | 0.511 | 0.078 | _ | _ | _ | _ | _ | _ | _ | _ | _ | _ | _ | _ | _ | _ | _ | _ |  |
|  |  |  |  |  |  |  |  |  |  |  | miR-125b-5p | Up | HR: 0.598 | 0.852 | 0.42 | 0.004 | _ | _ | _ | _ | _ | _ | _ | _ | _ | _ | _ | _ | _ | _ | _ | _ |  |
|  |  |  |  |  |  |  |  |  |  |  | miR-192-5p | Up | HR: 0.778 | 1.106 | 0.548 | 0.162 | _ | _ | _ | _ | _ | _ | _ | _ | _ | _ | _ | _ | _ | _ | _ | _ |  |
|  |  |  |  |  |  |  |  |  |  |  | miR-193b-3p | Up | HR: 1.11 | 1.575 | 0.783 | 0.558 | _ | _ | _ | _ | _ | _ | _ | _ | _ | _ | _ | _ | _ | _ | _ | _ |  |
|  |  |  |  |  |  |  |  |  |  |  | miR-221-3p | Up | HR: 0.878 | 1.249 | 0.618 | 0.47 | _ | _ | _ | _ | _ | _ | _ | _ | _ | _ | _ | _ | _ | _ | _ | _ |  |
|  |  |  |  |  |  |  |  |  |  |  | miR-27b-3p | Up | HR: 0.832 | 1.184 | 0.585 | 0.307 | _ | _ | _ | _ | _ | _ | _ | _ | _ | _ | _ | _ | _ | _ | _ | _ |  |
| 26 | Meijer, 2018 | Italy | Plasma | PDAC | _ | _ | 61.0 (43–75) | _ | 54 | _ | miR-181a-5p  decline | down | HR: 0.4 | 1.2 | 0.1 | 0.096 | median (SE):  6.4 (1.8) vs. 12.2 (1.9) | _ | _ | <0.005 | _ | _ | _ | _ | _ | _ | _ | _ | _ | _ | _ | _ |  |
| 27 | Nishiwada, 2020 | Japan | Tissue | PDAC | _ | _ | _ | _ | 157 | _ | 6-miRNA signature b | _ | HR: 1.78 | 2.73 | 1.16 | < 0.01 | _ | _ | _ | _ | _ | _ | _ | _ | _ | _ | _ | _ | _ | _ | _ | _ |  |
|  |  |  |  |  |  |  |  |  | 107 | _ | 6-miRNA signature b | _ | HR: 2.41 | 5.4 | 1.08 | 0.03 | _ | _ | _ | _ | _ | _ | _ | _ | _ | _ | _ | _ | _ | _ | _ | _ |  |
| 28 | Preis, 2011 |  | Tissue | PDAC | _ | noncancerous pancreatic specimens | _ | _ | 10 | 3 | miR-10b | Up | HR: 3.86 | 9.65 | 1.55 | 0.0032 | _ | _ | _ | _ | _ | _ | _ | _ | _ | _ | _ | _ | _ | _ | _ | _ |  |
|  |  |  |  |  |  |  |  |  |  |  | miR-10b | Up | HR: 5.1 | 20.7 | 1.25 | 0.02 | _ | _ | _ | _ | _ | _ | _ | _ | _ | _ | _ | _ | _ | _ | _ | _ |  |
| 29 | Schultz, 2012 | Denmark | Tissue | PDAC (160), A-AC (96) | _ | _ | _ | _ | 256 | _ | miR-675 | _ | HR: 1.13 | 1.22 | 1.06 | <0.001 | _ | _ | _ | _ | _ | _ | _ | _ | _ | _ | _ | _ | _ | _ | _ | _ |  |
|  |  |  |  |  |  |  |  |  |  |  | miR-148a* | _ | HR:0.90 | 0.96 | 0.84 | 0.002 | _ | _ | _ | _ | _ | _ | _ | _ | _ | _ | _ | _ | _ | _ | _ | _ |  |
|  |  |  |  |  |  |  |  |  |  |  | miR-146a | _ | HR:0.62 | 0.79 | 0.48 | <0.001 | _ | _ | _ | _ | _ | _ | _ | _ | _ | _ | _ | _ | _ | _ | _ | _ |  |
|  |  |  |  |  |  |  |  |  |  |  | miR-450b-5p | _ | HR:1.10 | 1.2 | 1.01 | 0.03 | _ | _ | _ | _ | _ | _ | _ | _ | _ | _ | _ | _ | _ | _ | _ | _ |  |
|  |  |  |  |  |  |  |  |  |  |  | miR-222 | _ | HR: 1.34 | 1.75 | 1.032 | 0.026 | _ | _ | _ | _ | _ | _ | _ | _ | _ | _ | _ | _ | _ | _ | _ | _ |  |
|  |  |  |  |  |  |  |  |  |  |  | miR-212 | _ | HR: 1.54 | 1.96 | 1.21 | <0.001 | _ | _ | _ | _ | _ | _ | _ | _ | _ | _ | _ | _ | _ | _ | _ | _ |  |
|  |  |  |  |  |  |  |  |  |  |  | miR-675 | _ | HR:1.08 | 1.14 | 1.02 | 0.009 | _ | _ | _ | _ | _ | _ | _ | _ | _ | _ | _ | _ | _ | _ | _ | _ |  |
|  |  |  |  |  |  |  |  |  |  |  | miR-148a* | _ | HR: 0.92 | 0.98 | 0.88 | 0.006 | _ | _ | _ | _ | _ | _ | _ | _ | _ | _ | _ | _ | _ | _ | _ | _ |  |
| 30 | Sun, 2016 | China | Serum | PDAC | _ | _ | _ | _ | 53 | _ | miR-124 | down | HR:2.87 | 4.53 | 1.126 | 0.002 | _ | _ | _ | _ | _ | _ | _ | _ | _ | _ | _ | _ | _ | _ | _ | _ |  |
| 31 | Yu, 2010 | Japan | Tissue | PC:  Adenocarcinoma(n=72)  Adenosquamous carcinoma(n=2) | _ | _ | 65.7 years (range, 36-86 years) | _ | 99 | _ | miR-200c | Up | HR:1.8 | 3.5 | 1 | 0.03 | _ | _ | _ | _ | _ | _ | _ | _ | _ | _ | _ | _ | _ | _ | _ | _ |  |
| 32 | Yu, 2010 | Japan | Tissue | PC:  Adenocarcinoma(n=72)  Adenosquamous carcinoma(n=2) | _ | _ | _ | _ | 80 | _ | miR-17-5p | Up | HR: 1.8 | 3.1 | 1 | 0.03 | _ | _ | _ | _ | _ | _ | _ | _ | _ | _ | _ | _ | _ | _ | _ | _ |  |
| 33 | Yu, 2017 | China | Serum | PDAC | _ | HC | 48.82 ± 9.75 | _ | 31 | 28 | miR-196a | Up | median: 6.6 vs 12.5 | 10.3 vs 15 | 2.3 vs 10 | 0.001 | _ | _ | _ | _ | _ | _ | _ | _ | _ | _ | _ | _ | _ | _ | _ | _ |  |
|  |  |  |  |  |  |  |  |  |  |  | miR-210 | Up | median: 11.7 vs 6.6 | 16.5 vs 7.9 | 6.8 vs 5.3 | 0.003 | _ | _ | _ | _ | _ | _ | _ | _ | _ | _ | _ | _ | _ | _ | _ | _ |  |
| 34 | Yu, 2019 | China | Tissue | PC | _ | normal adjacent tissues | 63.72 years (42-76 years) | _ | 60 | 60 | miR-539 | down | median: 20.48 vs 10.89 |  |  | 0.001 | _ | _ | _ | _ | _ | _ | _ | _ | _ | _ | _ | _ | _ | _ | _ | _ |  |
| 35 | Zhang, 2015 | China | Tissue | PDAC | _ | adjacent normal pancreas tissues | 57.9±9.85 | _ | 60 | 60 | miR-539 | down | HR: 1.728 | 2.712 | 1.225 | 0.025 | _ | _ | _ | _ | _ | _ | _ | _ | _ | _ | _ | _ | _ | _ | _ | _ |  |
|  |  |  |  |  |  | adjacent normal pancreas tissues | 57.9±9.85 | _ | 110 | 14 | miR-31 | _ | mean (SD): 10.25±16.49 |  | FDR:0.209 | 0.034 | _ | _ | _ | _ | _ | _ | _ | _ | _ | _ | _ | _ | _ | _ | _ | _ |  |
|  |  |  |  |  |  |  |  |  |  |  | miR-125b | _ | mean (SD): 18.45±11.02 |  | FDR:0.004 | 0.0002 | _ | _ | _ | _ | _ | _ | _ | _ | _ | _ | _ | _ | _ | _ | _ | _ |  |
|  |  |  |  |  |  |  |  |  |  |  | miR-186 | Up | mean (SD): 4.55±4.69 |  | FDR:0.036 | 0.005 | _ | _ | _ | _ | _ | _ | _ | _ | _ | _ | _ | _ | _ | _ | _ | _ |  |
|  |  |  |  |  |  |  |  |  |  |  | miR-221 | _ | mean (SD):  77.68±105.20 |  | FDR:0.036 | 0.005 | _ | _ | _ | _ | _ | _ | _ | _ | _ | _ | _ | _ | _ | _ | _ | _ |  |
|  |  |  |  |  |  |  |  |  |  |  | miR-224 | _ | mean (SD): 53.79±44.48 |  | FDR:0.015 | 0.001 | _ | _ | _ | _ | _ | _ | _ | _ | _ | _ | _ | _ | _ | _ | _ | _ |  |
|  |  |  |  |  |  |  |  |  |  |  | miR-326 | Up | mean (SD): 16.47±41.27 |  | FDR:<0.001 | <0.0001 | _ | _ | _ | _ | _ | _ | _ | _ | _ | _ | _ | _ | _ | _ | _ | _ |  |
|  |  |  |  |  |  |  | 57.3±9.57 | _ | 41 | 14 | miR-31 | _ | mean (SD): 5.57±8.56 |  | FDR:0.209 | 0.034 | _ | _ | _ | _ | _ | _ | _ | _ | _ | _ | _ | _ | _ | _ | _ | _ |  |
|  |  |  |  |  |  |  |  |  |  |  | miR-125b | _ | mean (SD):  29.43±20.81 |  | FDR:0.004 | 0.0002 | _ | _ | _ | _ | _ | _ | _ | _ | _ | _ | _ | _ | _ | _ | _ | _ |  |
|  |  |  |  |  |  |  |  |  |  |  | miR-186 | Up | mean (SD): 1.91±1.30 |  | FDR:0.036 | 0.005 | _ | _ | _ | _ | _ | _ | _ | _ | _ | _ | _ | _ | _ | _ | _ | _ |  |
|  |  |  |  |  |  |  |  |  |  |  | miR-221 | _ | mean (SD): 77.18±192.80 |  | FDR:0.036 | 0.005 | _ | _ | _ | _ | _ | _ | _ | _ | _ | _ | _ | _ | _ | _ | _ | _ |  |
|  |  |  |  |  |  |  |  |  |  |  | miR-224 | _ | mean (SD):  31.48±14.86 |  | FDR:0.015 | 0.001 | _ | _ | _ | _ | _ | _ | _ | _ | _ | _ | _ | _ | _ | _ | _ | _ |  |
|  |  |  |  |  |  |  |  |  |  |  | miR-326 | Up | mean (SD):  39.45±45.12 |  | FDR:<0.001 | <0.0001 | _ | _ | _ | _ | _ | _ | _ | _ | _ | _ | _ | _ | _ | _ | _ | _ |  |
|  |  |  |  |  |  |  | 57.8±9.75 | _ | 151 | 14 | mir-186 | Up | HR:1.655 | 2.41 | 1.137 | 0.009 | _ | _ | _ | _ | _ | _ | _ | _ | _ | _ | _ | _ | _ | _ | _ | _ |  |
|  |  |  |  |  |  |  |  |  |  |  | mir-224 | _ | HR:1.445 | 2.021 | 1.033 | 0.032 | _ | _ | _ | _ | _ | _ | _ | _ | _ | _ | _ | _ | _ | _ | _ | _ |  |
|  |  |  |  |  |  |  |  |  |  |  | mir-326 | Up | HR:0.476 | 0.715 | 0.317 | <0.001 | _ | _ | _ | _ | _ | _ | _ | _ | _ | _ | _ | _ | _ | _ | _ | _ |  |
|  |  |  |  |  |  |  |  |  |  |  | mir-221 | _ | HR: 1.432 | 2.036 | 1.007 | 0.046 | _ | _ | _ | _ | _ | _ | _ | _ | _ | _ | _ | _ | _ | _ | _ | _ |  |
| 36 | Zhu, 2013 | China | Tissue | PDAC | _ | nontumorous pancreatic tissues. | _ | _ | 94 | 16 | mir-141 | down | HR: 2.55 | 4.71 | 2.08 | 0.034 | _ | _ | _ | _ | _ | _ | _ | _ | _ | _ | _ | _ | _ | _ | _ | _ |  |
| 37 | Zhu, 2014 | China | Tissue | PDAC | _ | normal pancreatic tissue samples | _ | _ | 113 | 33 | mir-218 | down | RR: 2.119 | 2.499 | 1.505 | 0.017 | _ | _ | _ | _ | _ | _ | _ | _ | _ | _ | _ | _ | _ | _ | _ | _ |  |

**Supplementary Table 4.** Diagnostic Quality assessment

| Author, year | Selection | | | | Comparability | Exposure | | | Overall score |
| --- | --- | --- | --- | --- | --- | --- | --- | --- | --- |
|  | Case definition | Representativeness | Selection of Controls | Definition of Controls |  | Ascertainment of exposure | Same method of ascertainment | Non-Response rate |  |
| Xuan Zou, 2019 | * | * | * | * | ** | * | * | * | 9 |
| Zhenyong Wang, 2019 | * | * | * | * | ** | * | * | * | 9 |
| Fumitaka Ishige, 2020 | * | * | * | * | ** | * | * | * | 9 |
| So Nakamura, 2019 | * | * | C | B | ** | * | B | C | 5 |
| Dorbe, 2023 |  |  |  |  |  |  |  |  |  |
| Chen, 2014 |  |  |  |  |  |  |  |  |  |
| Shadan Ali, 2011 | * | * | C | B | ** | * | b | b | 5 |
| Barbara Alemar, 2015 | * | * | * | * | ** | * | * | * | 9 |
| Jia Chen, 2022 | * | * | * | * | ** | * | * | c | 8 |
| Gregory A, 2014 | B | * | * | * | ** | * | B | * | 7 |
| Ting Deng, 2016 | * | B | * | * | ** | * | * | * | 8 |
| Ali Seyed Salehi, 2022 | * | * | * | * | n/a | * | * | * | 7 |
| Eric J Duell, 2017 | * | B | * | * | ** | * | * | * | 8 |
| Lei Wang, 2021 | * | * | * | * | * | * | * | C | 7 |
| Maria Dobre, 2023 | * | * | * | * | ** | * | * | * | 9 |
| Takuma Goto, 2018 | * | * | * | * | ** | * | * | * | 9 |
| Neveen Abd El Moneim Hussein, 2016 | * | * | * | * | ** | * | * | * | 9 |
| Nigel B Jamieson, 2011 | * | * | C | B | ** | * | B | C | 5 |
| Jing Yu, 2021 | * | * | * | * | ** | * | * | * | 9 |
| Imeteyaz Ahmad Khan, 2021 | C | B | B | * | ** | A | * | * | 5 |
| N Le, 2019 | * | * | C | B | ** | * | B | C | 5 |
| Kote Nakamura, 2022 | * | b | * | * | ** | * | * | * | 8 |
| Yixing Wu, 2021 | b | B | B | * | ** | * | * | * | 6 |
| Qi Yu, 2017 | * | * | * | * | ** | * | * | * | 9 |
| Dawei Jiang, 2021 | * | * | * | * | ** | * | * | * | 9 |
| Quilan Chen, 2014 | * | * | * | * | ** | * | * | * | 9 |
| Rui Liu, 2012 | * | * | * | * | ** | * | * | * | 9 |
| Lissuly Guadalupe Álvarez-Hilario, 2023 | * | * | * | * | ** | * | * | * | 9 |
| Max Michael Traeger, 2018 | * | * | * | * | ** | * | * | * | 9 |
| Huilin Shao, 2021 | * | * | * | * | ** | * | * | * | 9 |
| Petra Vychytilova-Faltejskova, 2015 | * | * | * | * | ** | * | * | * | 9 |

**Supplementary Table 5.** Prognostic Quality assessment

| Author, year | Selection | | | | Comparability | Exposure | | | Overall score |
| --- | --- | --- | --- | --- | --- | --- | --- | --- | --- |
|  | Case definition | Representativeness | Selection of Controls | Definition of Controls |  | Ascertainment of exposure | Same method of ascertainment | Non-Response rate |  |
| Ziman Zhou, 2014 | * | * | * | b | ** | * | b | * | 7 |
| Ziman Zhu, 2013 | * | * | C | B | ** | * | B | C | 5 |
| Jun Zhou, 2021 | b | b | c | b | ** | * | b | b | 3 |
| Zheng-liang Zhang, 2015 | * | * | c | b | ** | * | b | c | 5 |
| Rui Zhang, 2014 | * | * | c | b | ** | * | b | c | 5 |
| Haibo Yu, 2018 | * | * | c | b | ** | * | B | C | 5 |
| Qi Yu, 2017 | * | * | * | * | ** | * | * | * | 9 |
| Jun Yu, 2010 | * | * | C | B | ** | * | B | c | 5 |
| Yan Chen, 2019 | * | * | C | b | ** | * | B | C | 5 |
| Jun Yu, 2010 | * | * | c | B | ** | * | B | C | 5 |
| Agnieszka Madro, 2023 | * | * | * | * | ** | * | b | * | 8 |
| Dawei Jiang, 2021 | * | * | * | * | ** | * | * | * | 9 |
| Zhenghai Bai, 2015 | * | * | * | * | * * | * | * | * | 9 |
| Nikhil T.Sebastian, 2021 | * | * | C | B | ** | * | B | c | 5 |
| S Caponi, 2012 | * | * | C | B | ** | * | B | C | 5 |
| Quilan Chen, 2014 | * | * | * | * | ** | * | * | * | 9 |
| Rui Liu, 2012 | * | * | * | * | ** | * | * | * | 9 |
| Sachiyo Kawamura, 2019 | * | * | C | B | ** | * | B | C | 5 |
| Elisa Giovannetti, 2012 | * | * | C | B | ** | * | B | C | 5 |
| Vince Kornél Grolmusz, 2018 | * | * | * | * | ** | * | B | C | 7 |
| Yongqiang Huam 2017 | * | * | * | * | ** | * | * | * | 9 |
| Jinghua Liu, 2016 | * | * | C | B | ** | * | B | C | 5 |
| Jun Ma, 2017 | * | * | * | * | ** | * | * | * | 9 |
| Lissuly Guadalupe Álvarez-Hilario, 2023 | * | * | * | * | ** | * | * | * | 9 |
| Lucie Jiraskova, 2019 | * | * | C | B | ** | * | B | C | 5 |
| Petr Karasek, 2018 | * | * | b | * | ** | * | * | * | 8 |
| Xiangyu Kong, 2010 | * | * | * | * | ** | * | * | * | 9 |
| Hirokazu Kubo, 2019 | * | * | C | B | ** | * | B | C | 5 |
| ChongLek Lee, 2013 | * | * | C | B | ** | * | B | C | 5 |
| Laura L Meijier, 2018 | * | * | * | * | ** | * | * | * | 9 |
| Max Michael Traeger, 2018 | * | * | * | * | ** | * | * | * | 9 |
| Satoshi Nishiwada, 2020 | B | B | C | B | ** | * | B | C | 3 |
| Huilin Shao, 2021 | * | * | * | * | ** | * | * | * | 9 |
| Meir Preis, 2011 | * | * | C | B | ** | * | B | C | 5 |
| Nicolai A, 2012 | * | B | C | B | ** | * | B | C | 4 |
| BoLin Sun, 2016 | * | * | * | * | ** | * | * | * | 9 |
| Petra Vychytilova-Faltejskova, 2015 | * | * | * | * | ** | * | * | * | 9 |

**Supplementary Figure 1.** Diagnostic accuracy meta-analysis of the microRNAs in different sample types.


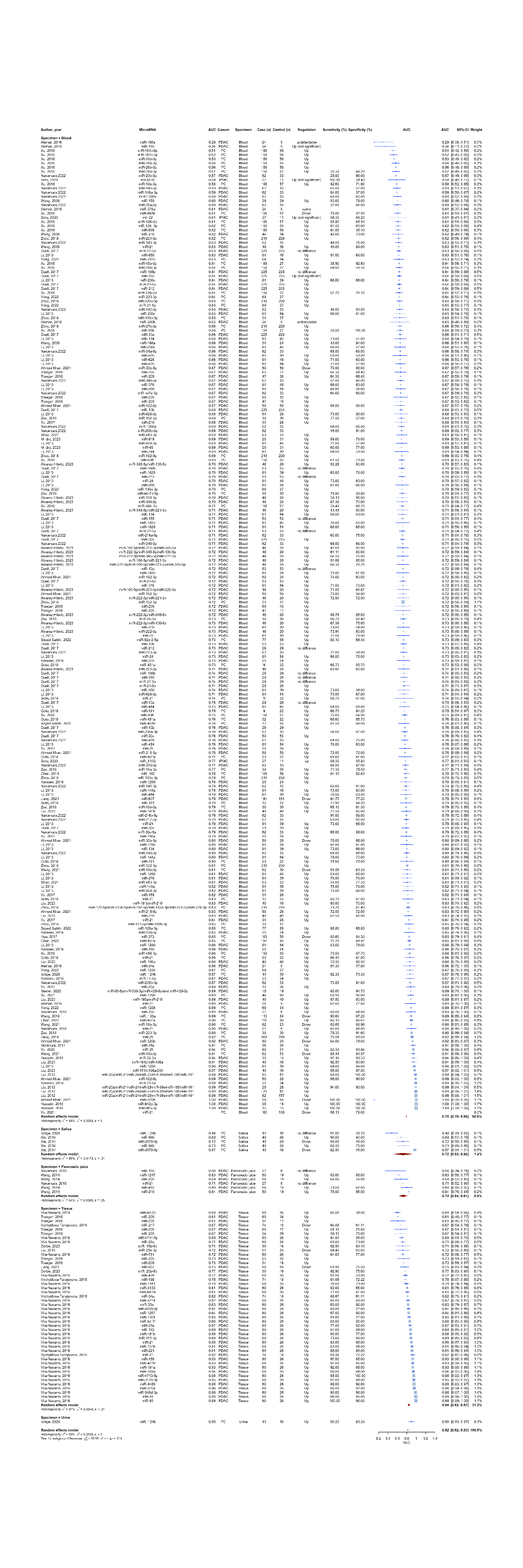

Supplement: Supplementary file 1 — Data S1. [file JCMM-29-e70337-s001.docx]
